# Characteristics of the Conjugative Transfer System of the IncM Plasmid pCTX-M3 and Identification of Its Putative Regulators

**DOI:** 10.1128/JB.00234-18

**Published:** 2018-08-24

**Authors:** Michał Dmowski, Marcin Gołębiewski, Izabela Kern-Zdanowicz

**Affiliations:** a Institute of Biochemistry and Biophysics, Department of Microbial Biochemistry, Polish Academy of Sciences, Warsaw, Poland; Princeton University

**Keywords:** IncM group, conjugative transfer, plasmid analysis, plasmid mobilization

## Abstract

Plasmid conjugative transfer systems comprise type IV secretion systems (T4SS) coupled to DNA processing and replication. The T4SSs are divided into two phylogenetic subfamilies, namely, IVA and IVB, or on the basis of the phylogeny of the VirB4 ATPase, into eight groups. The conjugation system of the IncM group plasmid pCTX-M3, from Citrobacter freundii, is classified in the IVB subfamily and in the MPF_I_ group, as are the conjugation systems of IncI1 group plasmids. Although the majority of the conjugative genes of the IncM and IncI1 plasmids display conserved synteny, there are several differences. Here, we present a deletion analysis of 27 genes in the conjugative transfer regions of pCTX-M3. Notably, the deletion of either of two genes dispensable for conjugative transfer, namely, *orf35* and *orf36*, resulted in an increased plasmid mobilization efficiency. Transcriptional analysis of the *orf35* and *orf36* deletion mutants suggested an involvement of these genes in regulating the expression of conjugative transfer genes. We also revised the host range of the pCTX-M3 replicon by finding that this replicon is unable to support replication in Agrobacterium tumefaciens, Ralstonia eutropha, and Pseudomonas putida, though its conjugation system is capable of introducing plasmids bearing *oriT*_pCTX-M3_ into these bacteria, which are representatives of *Alpha*-, *Beta*-, and Gammaproteobacteria, respectively. Thus, the conjugative transfer system of pCTX-M3 has a much broader host range than its replicon.

**IMPORTANCE** Horizontal gene transfer is responsible for rapid changes in bacterial genomes, and the conjugative transfer of plasmids has a great impact on the plasticity of bacteria. Here, we present a deletion analysis of the conjugative transfer system genes of the pCTX-M3 plasmid of the IncM group, which is responsible for the dissemination of antibiotic resistance genes in Enterobacteriaceae. We found that the deletion of either of the *orf35* and *orf36* genes, which are dispensable for conjugative transfer, increased the plasmid mobilization efficiency. Real-time quantitative PCR (RT-qPCR) analysis suggested the involvement of *orf35* and *orf36* in regulating the expression of transfer genes. We also revised the host range of pCTX-M3 by showing that its conjugative transfer system has a much broader host range than its replicon.

## Abstract (precis)

Horizontal gene transfer is responsible for rapid changes in bacterial genomes, and the conjugative transfer of plasmids has a great impact on the plasticity of bacteria. Here, we present a deletion analysis of the conjugative transfer system genes of the pCTX-M3 plasmid of the IncM group, which is responsible for the dissemination of antibiotic resistance genes in Enterobacteriaceae. We found that the deletion of either of the *orf35* and *orf36* genes, which are dispensable for conjugative transfer, increased the plasmid mobilization efficiency. Real-time quantitative PCR (RT-qPCR) analysis suggested the involvement of *orf35* and *orf36* in regulating the expression of transfer genes. We also revised the host range of pCTX-M3 by showing that its conjugative transfer system has a much broader host range than its replicon.

## INTRODUCTION

Conjugative transfer is a prevalent phenomenon among bacteria; this phenomenon is crucial for horizontal gene transfer in the biosphere and is a major contributor to the rapid variability of bacterial genomes. In the process of conjugative transfer, DNA (a conjugative plasmid, a conjugative transposon, or an integrative conjugative element [ICE]) is transferred from a donor to a recipient cell after physical contact between the cells is established. The process may be regarded as a combination of DNA processing functions coupled to a type IV secretion system (T4SS; also known as mating pair formation [Mpf]) by a dedicated protein (coupling protein [CP]). The DNA processing functions are provided by the DNA transfer and replication (Dtr) system, also called the relaxosome complex (1). The T4SSs of Gram-negative bacteria are classified into two large phylogenetic groups, namely, IVA and IVB: the Agrobacterium tumefaciens VirB/D4 secretion system and the conjugation systems of the IncF and IncP plasmids are classified as type IVA (T4ASS) (2, 3), whereas type IVB (T4BSS) is represented by secretion systems found in Legionella pneumophila (Dot/Icm) and in other important pathogens (4, 5). The majority of the Dot/Icm proteins share homology with the constituents of the conjugation system of the R64 plasmid of the IncI1 group. Despite an increasing amount of information becoming available in recent years on the organization and regulation of T4BSSs, they are still less thoroughly characterized than T4ASSs.

The canonical T4SS is represented by the Agrobacterium tumefaciens VirB/D4 secretion system responsible for transfer DNA (T-DNA) transfer to plant cells during infection. This T4SS consists of 11 proteins (VirB1 to VirB11) and the coupling protein VirD4 (for review, see references 5 to 8). The translocation channel comprises the VirB3, B6, B7, B8, B9, and B10 proteins. In the translocation channel, three components form the core channel complex in the outer membrane (OM), also called outer membrane complex (OMC): VirB9, the pore-forming protein; VirB7, a small lipoprotein; and VirB10, the protein spanning both the inner membrane (IM) and the outer membrane (OM). Interactions of the OMC with the inner membrane complex (IMC), which comprises the VirB3, VirB6, and VirB8 proteins, and with the ATPases VirB4 and VirB11 result in the formation of a pore. The extracellular structure important for the establishment of contact between mating cells, namely, the T-pilus, is composed of the major subunit VirB2 and the minor component VirB5 localized at the tip. VirB3, the least-characterized Mpf component, is also necessary for T-pilus assembly. Finally, VirB1 shows homology to a lytic transglycosylase that cleaves peptidoglycan (3, 9). The system is energized by three cytoplasmic ATPases: VirB4, VirB11, and the coupling protein VirD4. Of all of the Vir proteins listed above, VirB4 is the only component present in every T4SS described so far (10). The universal presence of VirB4 enabled all known Mpfs of both Gram-negative and Gram-positive bacteria and archaea to be divided into eight groups on the basis of VirB4 phylogeny (11). The A. tumefaciens VirB/D4 system and the conjugation system of the IncP plasmids are now classified in the MPF_T_ group (11). The conjugation system of the F plasmid, one of the most well-known plasmids, belongs to the MPF_F_ group (11). In addition, the IncI1 plasmid R64 codes for TraU, which is a distant VirB4 homologue, and constitutes the prototype of the MPF_I_ group. The R64 conjugative transfer system is encoded by 22 transfer genes, namely, *traE-traY*, three *trbA-C* genes, and the *nuc* gene, 16 of which have been shown to be indispensable for conjugation (12). The homology of R64 to the VirB/D4 system of A. tumefaciens is rather low. However, TraO displays homology to VirB10 (13), and TraM is distantly homologous to VirB8, TraJ to the VirB11 ATPase, and TraQ and TraR to the pilin subunit VirB2 (11).

Another plasmid encoding an MPF_I_ conjugation system that displays homology to the T4BSS systems of the IncI1 plasmids is pCTX-M3 (accession no. AF550415), a member of the IncM incompatibility group (14). Plasmid pCTX-M3 was isolated from a clinical Citrobacter freundii strain in Poland in 1996 as a vector of the *bla*_CTX-M-3_ gene (14, 15). It is noteworthy that members of the IncM group are closely related not only to each other but also to plasmids of the IncL group (16), with which they were earlier classified jointly as the IncL/M group (17). IncL and IncM plasmids are widespread in Enterobacteriaceae (18) and are responsible for the dissemination of antibiotic resistance genes. These genes include *bla*_CTX-M-3_, which encodes an extended spectrum β-lactamase, *bla*_NDM-1_, which encodes a metallo-β-lactamase, *bla*_OXA-48_, which encodes a carbapenem-hydrolyzing enzyme (19, 20), *bla*_KPC-4_, which codes for the Klebsiella pneumoniae carbapenemase (21), *bla*_IMP-4_, which codes for imipenemase (22), and the aminoglycoside resistance gene *armA* (23).

**FIG 1:**
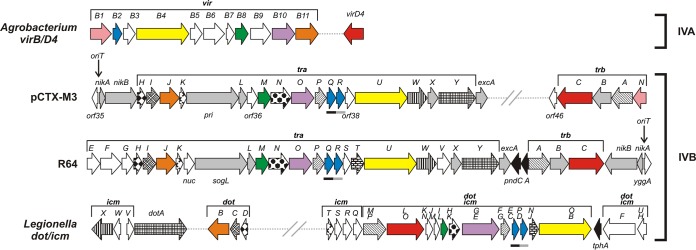
Gene organization of type IV secretion systems: IVA, the A. tumefaciens virB/*virD4* region, and IVB, the transfer regions of pCTX-M3, R64, and the *dot* or *icm* region of L. pneumophila. Open reading frames (ORFs) are represented by arrows indicating their orientation. The ORFs homologous in all systems are shown in the same colors (except black, gray, and white). The ORFs homologous in the type IVB systems are indicated by identical patterns. The ORFs homologous in both plasmids are shown in gray, and those specific for either system are shown in white. The bars under the genes (pCTX-M3, R64, and *dot* or *icm*) indicate homology of single genes. The lines above the genes indicate the gene designation, and the corresponding letter is indicated above each gene. Dotted lines indicate small regions of unrelated genes, and line breaks indicate large gaps. The ORFs not related to the T4SS are shown as black arrows.

The pCTX-M3 plasmid can be transferred by approximately 10% of cells in an Escherichia coli donor population under optimal conditions (14). Bacteria bearing pCTX-M3 can also conjugate in liquid culture; however, in contrast to IncI1 plasmids, which require type IV pili for conjugation in liquid media, pCTX-M3 does not encode additional pili (14, 24, 25). The conjugative transfer genes of pCTX-M3 are localized in two separate regions with predicted operon structures, namely, *tra* and *trb* (14), and these genes do not exhibit substantial sequence similarity to genes with ascribed functions available in public databases apart from those encoded by IncI1 and IncL/IncM plasmids (Fig. 1). The *tra* and *trb* genes of IncL/IncM plasmids such as pCTX-M3 encode proteins that exhibit 39% to 65% similarity to those encoded by IncI1 plasmids (such as R64 and ColIb-P9) (Table 1); however, a number of genes from each system do not have counterparts in the other system (14). The pCTX-M3 plasmid can mobilize plasmids that contain the heterologous *oriT*_ColIb-P9_ (from the IncI1 plasmid ColIb-P9), and plasmids with *oriT*_pCTX-M3_ can be mobilized by a ColIb-P9-derived plasmid (14); both of these mobilizations occur at low frequencies.

**TABLE 1:** Bioinformatics analysis of the predicted proteins encoded in the transfer region of pCTX-M3

Protein	Size (aa)	Mol wt (kDa)^*a*^	pI^*a*^	SP^*b*^	TMH^*c*^	Motif found/putative function^*d*^	Homologues in IncI1 plasmid and Legionella pneumophila *dot* or *icm* (aa [% aa sequence identity/% aa sequence similarity])^*e*^
Orf35	121	9.0	13.57	No	No		6–108 (32/53) to YggA of R64
NikA	105	11.6	9.41	No	No	Helix-turn-helix motif/nickase accessory protein	3–101 (30/62) to NikA of R64
NikB	658	75.1	9.53	No	No	Relaxase domain/nickase (Rlx)^*f*^	1–388 (31/48) to NikB of ColIb-P9
TraH	166	18.7	9.77	Yes	1	Lipoprotein^*g*^/CTS	70–157 (45/65) to TraH of ColIb-P9
TraI	259	29.2	9.33	Yes	1	NA/CTS	29–258 (48/65) to TraI of ColIbP9; 3–258 (28/46) to DotC of L. pneumophila
TraJ	387	43.3	6.05	No	1	GSPIIE domain with Walker motifs A and B/ATPase VirB11-like (TivB11)^*f*^	11–369 (40–63) to TraJ of ColIb-P9; 11–377 (31/51) to DotB of L. pneumophila
TraK	86	10.3	10.55	No	2		6–82 (41/61) to TraK of ColIb-P9; 6–80 (25/45) to IcmT of L. pneumophila
Pri	1070	117.6	5.00	No	No	NA/primase (Pri)^*f*^	4–556 (30/45) and 389–954 (23/39) to SogL of R64
TraL	170	17.9	6.27	Yes	1		14–114 (33/58) to TraL of ColIb-P9
Orf36	221	25.0	9.41	No	3		None
TraM	260	29.5	5.72	No	1	NA/VirB8-like (TivB8)^*f*^	53–254 (26/53) to TraM of ColIb-P9; 73–257 (31/53) to DotI (IcmL) of L. pneumophila
TraN	383	40.7	5.97	Yes	1	NA/CTS	119–382 (45/58) to TraN of ColIb-P9; 47–381 (29/44) to DotH (IcmK) of L. pneumophila
TraO	449	47.6	5.90	No	1	VirB10 domain/CTS VirB10-like (TivB10)^*f*^	4–419 (30/45) to TraO of ColIb-P9; 208–390 (41/50) to DotG (IcmE) of L. pneumophila
TraP	234	24.7	9.20	No	1	NA/CTS	22–234 (23/40) to TraP of ColIb-P9
TraQ	176	18.5	8.49	No	3	NA/pilin	32–174 (35/54) to TraQ of ColIb-P9; 5–172 (25/43) to DotE (IcmC) of L. pneumophila
TraR	129	13.5	9.72	Yes	3	NA/pilin	12–127 (26/47) to TraR of ColIb-P9
Orf38	164	18.9	6.96	No	no		None
TraU	1016	114.1	6.24	No	3	Walker motifs A and B/ATPase VirB4-like (TivB4)^*f*^	1–1,006 (35/56) to TraU of ColIb-P9; 27–1,008 (28/47) to DotO (IcmB) of L. pneumophila
TraW	402	43.3	8.58	Yes	3		14–401 (36/55) to TraW of ColIb-P9
TraX	216	24.1	9.23	No	3		92–201 (31/48) to TraX of ColIb-P9
TraY	726	78.3	5.58	Yes	7	NA/entry exclusion system	4–725 (37/55) to TraY of ColIb-P9; 4–217 (25/45) and 526–683 (25/46) to DotA of L. pneumophila
ExcA	217	25.4	9.28	Yes	3	NA/entry exclusion system	55–132 (31/47) to ExcA of ColIb-P9
Orf46	169	19,2	9.79	No	No	Transcriptional regulator of ROS/MUCR superfamily	None
TrbC	695	79.5	5.08	No	3	Walker motifs A and B/CP (Cpl)^*f*^	29–609 (34/52) to TrbC of ColIb-P9; 107–599 (30/50) to DotL (IcmO) of L. pneumophila
TrbB	338	37.4	9.10	No	1	Thioredoxin-like domain/NA	121–256 (37/47) to TrbB of ColIb-P9
TrbA	435	49.1	6.43	No	3	NA/CP (Cpl)^*f*^ complex	13–124 (41/57) to TrbA of ColIb-P9; 80–368 (24/44) to DotM (IcmP) of L. pneumophila
TrbN	131	14.7	9.14	No	No	Lytic transglycosylase signature/Slt^*f*^	None

a Calculated with the use of the ProtParam tool of Expasy.

b SP, signal peptide determined with the use of SignalP v.2.0.

c TMH, transmembrane helices determined with the use of TMPred.

d Determined with MotifScan. NA, not available; CP, coupling protein; CTS, core transmembrane subcomplex.

e Determined with BLASTp.

f Protein name according to reference 51.

g Determined with LipoP.

Here, we present a deletion analysis of the *tra* and *trb* genes potentially involved in pCTX-M3 conjugative transfer. We found that the deletion of either of the *orf35* and *orf36* genes, both of which are dispensable for pCTX-M3 transfer, increases the mobilization efficiency of *oriT*_pCTX-M3_-bearing plasmids into E. coli and A. tumefaciens. The deletion of these genes also affected the transcription of other conjugative transfer genes. In addition, we verified the host range of the pCTX-M3 conjugation system and found that the host range of its replicon, reported previously to comprise *Alpha*-, *Beta*-, and Gammaproteobacteria (26), is in fact much narrower than previously believed and is limited to Enterobacteriaceae.

## RESULTS AND DISCUSSION

### Organization of the regions coding for the conjugative transfer systems of the pCTX-M3 and R64 plasmids.

In pCTX-M3, both the *tra* and *trb* regions coding for the conjugative transfer system display extensive conservation of synteny with the conjugation system genes of the IncI1 plasmids R64 and ColIb-P9 (Fig. 1) (14). However, there are certain differences. pCTX-M3 has no homologues of the *traEFG*, *traST*, and *traV* genes. Neither *traEFG* nor *traS* is required for the conjugative transfer of R64, whereas *traT* and *traV* are indispensable (12). Moreover, in pCTX-M3, the *nuc* gene, which encodes a nuclease, is located at a distance from the *tra* region. In addition, the single *orf38* located between *traR* and *traU* replaces *traST*, while *orf36*, which is found only in IncL and IncM plasmids, separates *traL* and *traM*. Furthermore, the *trbN* gene of pCTX-M3, which encodes a putative lytic transglycosylase, has no homologues in R64. The homologue of *orf46* is also absent in R64. However, the major difference between these plasmids concerns the position of their *oriT* regions with the *nikAB* genes. In R64, *oriT* lies far from the *tra* genes in a tail-to-tail orientation with respect to the *trb* operon. In pCTX-M3, the *oriT* region, along with the *nikAB* genes, is situated immediately upstream of the *tra* genes, and the *nikAB* and *tra* genes are predicted to constitute a single operon (Fig. 1) (14).

### Identification of genes necessary for conjugation.

**FIG 2:**
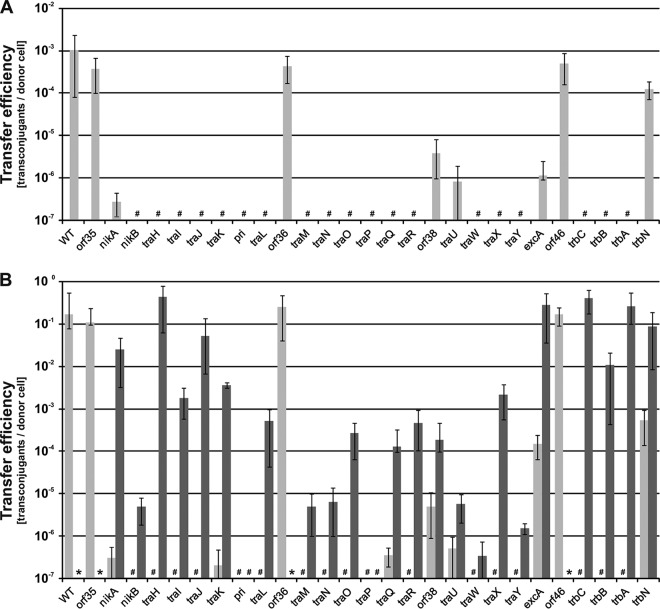
Deletion analysis of the pCTX-M3 transfer region. (A) Conjugative transfer efficiency of pCTX-M3 derivatives in liquid mating. (B) Conjugative transfer efficiency of pCTX-M3 derivatives (light gray) and pCTX-M3 derivatives complemented with plasmids bearing the relevant genes (dark gray) in filter mating. #, undetectable transfer (<10^−7^); *, complementation not analyzed. Each result is the mean from at least three experiments. Error bars indicate the SDs.

A systematic deletion analysis was performed for the pCTX-M3 genes in the *tra* and *trb* regions. For this purpose, a collection of 27 derivatives with a deletion in each of the genes in the *tra* and *trb* regions, as well as *orf35* from the pCTX-M3 leading region and *orf46*, which is located downstream of *trbC*, was constructed (see Table S1 in the supplemental material) by replacing a given gene with the *cat* gene (27). To avoid a difference in expression levels depending on the position of the gene in the operon, the *cat* gene with its own promoter sequence was inserted in the opposite orientation to that of the *tra* or *trb* genes. For each pCTX-M3 deletion derivative, the frequency of conjugative transfer (conjugation efficiency) from E. coli BW25113 cells to the recipient E. coli JE2571Rif^r^ cells, both in liquid and on filters, was determined (Fig. 2A). Liquid mating occurred at a lower efficiency than filter mating; therefore, further conjugative transfer analysis of the pCTX-M3 derivatives was performed using filter mating only. All the plasmids displayed a diminished or completely inhibited conjugative transfer ability except for pCTX-M3*orf35*::*cat*, pCTX-M3*orf36*::*cat*, and pCTX-M3*orf46*::*cat*; these plasmids showed the same conjugation efficiency as the parental pCTX-M3 plasmid, which was approximately 10^−1^ for filter mating (Fig. 2B). We therefore concluded that *orf35*, *orf36*, and *orf46* are dispensable for conjugative transfer.

### Complementation of the deleted *tra* and *trb* genes.

To exclude the possibility that the reduced conjugative transfer of the pCTX-M3 deletion derivatives was caused by a polar effect, each mutated plasmid was complemented with an appropriate gene cloned into pMT5 or pAL3 under the control of the *P_lac_* promoter (Table S1). For the majority of the pCTX-M3 derivatives, the complementation fully or at least partially restored the conjugation efficiency (10^−4^ to 10^−1^ per donor cell). However, for the *nikB*, *traM*, *traN*, *traU*, *traW*, and *traY* deletion derivatives, the presence of the complementing gene rescued the conjugation efficiency only to levels less than 10^−5^; for *traP* and *pri*, the complementation had no detectable effect (Fig. 2B). Although several independent *traP* and *pri* deletion mutants were investigated, none regained the conjugative transfer ability upon complementation with pMT5*traP* or pAL*pri*, respectively.

**FIG 3:**
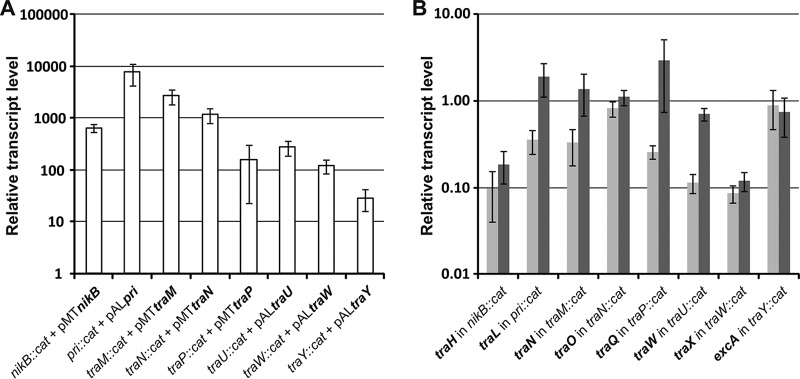
Transcript levels of genes in the pCTX-M3 plasmid deletion derivatives. (A) Transcript levels of the complementing genes (in bold) in the respective deletion derivatives. (B) Transcript levels of genes (in bold) located directly downstream of the deleted gene in the respective pCTX-M3 deletion derivatives (light gray) complemented with the appropriate plasmid (dark gray). Each result is the mean value from biological triplicates normalized to the transcript level of the appropriate gene in the BW25113(pCTX-M3) strain. Error bars indicate the SDs.

In R64, the disruption of *nikB*, which encodes nickase (relaxase), abolished conjugative transfer; however, *nikB* can be complemented well (28). Furthermore, the disruption of *traM*, *traN*, *traW*, and *traY* was detrimental to the transfer of R64, but these genes were able to be complemented by plasmids expressing the relevant gene to the wild-type (WT) transfer efficiency or, for *traY*, 10× lower than the WT transfer efficiency (12). In R64, disruptions of *traU* are detrimental to transfer. Similar to the effect of limited complementation of pCTX-M3*traU*::*cat*, the transfer efficiency of R64 that expressed a *traU* gene disrupted close to its start site but that was complemented by a plasmid bearing *traU* was 9 × 10^−7^ (12). Taking into account the differences in complementation between the respective mutants of pCTX-M3 and R64, the *nikB*, *traM*, *traN*, *traW*, *traY*, *pri*, *traP*, and *traU* mutants of pCTX-M3 were analyzed further. First, the expression of the complementing genes was verified by real-time quantitative PCR (RT-qPCR). The transcript levels of all these genes were at least 10-fold higher than those found in the strain bearing pCTX-M3 (Fig. 3A).

Given that a polar effect of the deletion of those genes was not strictly excluded, the transcription of genes located directly downstream of the target genes was quantified by RT-qPCR (Fig. 3B). Indeed, the transcript levels of each of the downstream genes analyzed, except for those of the *traN*::*cat* and *traY*::*cat* derivatives, were lower than those found in the strain bearing the intact pCTX-M3 (Fig. 3B). The complementation of the analyzed deletions with the appropriate genes restored the expression of the downstream genes to the levels observed in the strain with pCTX-M3 for the *pri*, *traM*, and *traP* deletion mutants and nearly restored expression for the *traU* deletion mutant, but complementation had only a small effect on the *nikB*::*cat* and *traW*::*cat* mutants (Fig. 3B). In the case of the *traY*::*cat* mutant, where no decrease in the expression of the downstream gene was found, the complementation had no effect on the transcript level of the downstream gene.

**FIG 4:**
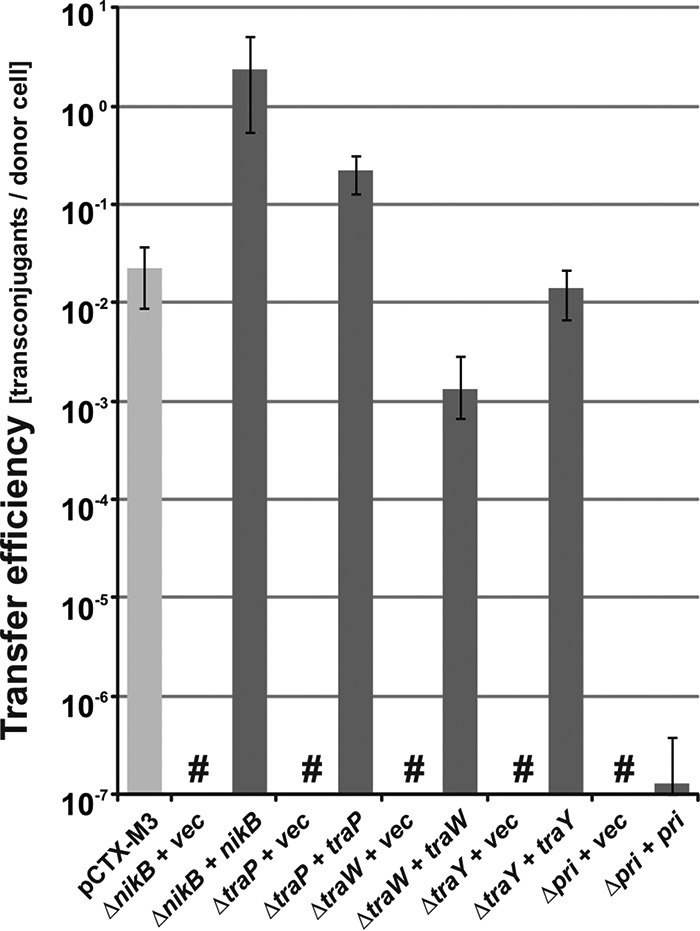
Conjugative transfer efficiency of pCTX-M3 derivatives with the *cat* gene eliminated. Conjugative transfer efficiency of pCTX-M3 (light gray) and pCTX-M3 derivatives (pCTX-M3Δ*nikB*, pCTX-M3Δ*traP*, pCTX-M3Δ*traW*, pCTX-M3Δ*traY*, and pCTX-M3Δ*pri*) complemented with plasmids bearing the appropriate genes (dark gray) in filter mating. vec, empty vector; #, undetectable transfer (<10^−7^). Each result is the mean from at least five experiments. Error bars indicate the SDs.

The results so far showed that five deletion mutants of pCTX-M3 behaved in an unexpected manner: *traP* and *pri*, which could not be complemented; *traY*, where the complementation was very poor; and *nikB* and *traW*, where the downstream genes, *traH* and *traX*, respectively, showed low expression in addition to poor complementation. To determine whether the behavior of these genes was due to the deletion of the genes in question or was connected with the presence of the *cat* gene in their loci, the *cat* gene was eliminated from the mutants with the use of the pCP20-encoded FLP recombinase. The ability of the constructed plasmids (pCTX-M3Δ*traP*, pCTX-M3Δ*pri*, pCTX-M3Δ*traW*, pCTX-M3Δ*traY*, and pCTX-M3Δ*nikB*) to undergo conjugative transfer was tested after complementation with a plasmid bearing the corresponding gene (Fig. 4). All the pCTX-M3 derivatives lacking both the gene of interest and the *cat* gene were transferred by conjugation when the missing gene was delivered in *trans*: the transfer efficiencies of the Δ*nikB*, Δ*traP*, Δ*traW*, and Δ*traY* mutated plasmids were greater than 10^−3^ transconjugants per donor cell (Fig. 4). Therefore, we conclude that the lack of complementation of the previously described plasmids with mutated *nikB*, *traP*, *traW*, and *traY* was connected to the presence of the *cat* gene in the loci of the affected genes and was corrected by *cat* elimination.

However, the transfer efficiency of pCTX-M3Δ*pri* after complementation was low (approximately 2 × 10^−7^). The results obtained for the *pri* deletion in pCTX-M3 differ from those for the *sog* deletion in R64, where the deletion resulted in only a small drop in the transfer efficiency, from 10^−2^ to 10^−3^ (29). The *sog* gene encodes two proteins, the SogL primase (1,255 amino acids [aa]), which generates RNA primers for plasmid replication, and the shorter SogS (844 aa), which is a product of translational reinitiation within the *sog* reading frame (30). It was speculated that Sog proteins create a complex with DNA, coating the transferred single DNA strand to protect and stabilize it (31). Both proteins are transported from the donor to the recipient cell during conjugation, and the transport has been shown to rely on the *pil* genes encoding thin pili (32), which are absent in pCTX-M3 (14). The *pri* gene of pCTX-M3 also has the potential to encode two proteins, a primase (1,070 aa) and a putative 689-aa protein comprising the C-terminal moiety of the primase. The high level of the *pri* transcript (Fig. 3A) might result in the production of a large amount of these two proteins, which can coat the single-stranded DNA (ssDNA) of the transferred plasmid and block the transporter. However, the reason for the lack of complementation of the *pri*-deficient pCTX-M3 is highly speculative, and the pCTX-M3 *pri* gene therefore needs further study.

The results presented above demonstrate that though the disruptions in the majority of the *tra* and *trb* genes of the pCTX-M3 plasmid, which were performed via *cat* insertion, do not prevent the expression of a functional conjugative transfer system, in some cases, the deletion of the inserted *cat* gene was required. In these cases, the observed decreased transcript levels and lower conjugation efficiencies of the mutated plasmids even in the presence of the appropriate complementing genes suggest a defective regulation of gene expression. The mechanism of the regulation of the pCTX-M3 *tra* and *trb* operons is unknown and needs further research.

### Putative roles of the *tra* and *trb* genes of pCTX-M3. (i) Putative components of the T4CP subcomplex.

The analysis of the *trb* region of pCTX-M3 showed that the deletion of either *trbA*, *trbB*, or *trbC* abolished conjugation. In R64, the *trbA* and *trbC* genes were found to be indispensable for conjugative transfer, while *trbB* was required for a high transfer efficiency (33). TrbC_pCTX-M3_, with its ATPase Walker motifs A and B, is homologous to TrbC_R64_ and to DotL (IcmO) of L. pneumophila, which acts as a type IV coupling protein (T4CP) (Table 1). In the Dot/Icm system, DotL forms the T4CP subcomplex along with other proteins that have no CP function: two inner membrane proteins, namely, DotM (IcmP) and DotN (IcmJ), and the secretion adapter proteins IcmS and IcmW (34). The DotM homologue of pCTX-M3 is encoded by the *trbA* gene. Homologues of TrbB_pCTX-M3_, except for the TrbB proteins encoded by IncI1 plasmids, are putative disulfide bond isomerases. Therefore, the *trbC* gene of pCTX-M3 is likely to encode the coupling protein, while *trbA* codes for an element of the T4CP subcomplex, whose other components are to be characterized.

### (ii) Putative components of the transmembrane subcomplex.

The *traN* gene of both R64 (12) and pCTX-M3 encodes a homologue of DotH (IcmK) of L. pneumophila. The proper localization of DotH in the OM is assisted by the lipoproteins DotC and DotD, which together form a pore similar to that formed by the VirB7/VirB9 proteins of A. tumefaciens (13). DotC, DotD, and DotH, along with the IM proteins DotF (IcmG) and DotG (IcmE), have been found to form the core transmembrane subcomplex that bridges the IM and the OM in L. pneumophila (13, 35). A homologue of L. pneumophila DotC, encoded by *traI*, is indispensable for the conjugative transfer of pCTX-M3, while the disruption of *traI*_R64_ led only to a reduction in the transfer efficiency (12). In pCTX-M3 and R64, TraH is a homologue of DotD of L. pneumophila; however, in contrast to the deletion of *traH*_R64_ (12), the disruption of *traH*_pCTX-M3_ abolishes the transfer of pCTX-M3. The putative localization of TraH_pCTX-M3_ in the cell membrane is supported by the presence of a predicted signal peptide and a lipid attachment motif in its sequence (Table 1). Distant homologues of DotF of L. pneumophila are TraP_pCTX-M3_ and TraP_R64_, which are necessary for the conjugative transfer of both plasmids. In the TraP_pCTX-M3_ sequence, a single transmembrane helix was found, suggesting IM localization (Table 1). DotG of L. pneumophila shares homology with TraO_R64_ (13) and TraO_pCTX-M3_ (Table 1) homologues of VirB10 of A. tumefaciens. Interestingly, the deletion of the *traO* gene abolished the conjugative transfer of pCTX-M3, while in R64, *traO* deletion only reduced the transfer efficiency (12). Therefore, we propose that TraH, TraI, TraN, TraO, and TraP of both pCTX-M3 and R64 are components of the core transmembrane subcomplex. The different consequences of the deletion of *traH*, *traI*, or *traO* on the conjugative transfer of pCTX-M3 and R64 raise the possibility that the compositions of the core transmembrane subcomplexes encoded by these two plasmids are also different.

### (iii) Putative functions of other pCTX-M3-encoded proteins.

The *nikA* and *nikB* genes encode components of the nickase complex (an auxiliary protein and a relaxase, respectively). The deletion of these genes completely abolishes the conjugative transfer of pCTX-M3 and R64 (36). The *traJ* gene encodes an ATPase homologous to VirB11 of A. tumefaciens and DotB of L. pneumophila (37) (Table 1). Its deletion abolishes the conjugative transfer of pCTX-M3, while in R64, *traJ* deletion only reduced the transfer efficiency (12). In turn, TraK_R64_ and TraK_pCTX-M3_ (Table 1) are homologues of the IcmT protein of L. pneumophila, whose function is unknown (37). TraM_R64_ and TraM_pCTX-M3_ display homology with DotI (IcmL) of L. pneumophila and with VirB8 of A. tumefaciens (11, 38). The *traU* gene encodes a putative ATPase that is a homologue of DotO (IcmB) of L. pneumophila (37, 39). The encoded protein is also distantly homologous to VirB4 (11), which is involved in pilus assembly (40) and is essential for the virulence of Agrobacterium (41).

The *traR* and *traQ* genes, which are indispensable for the conjugative transfer of pCTX-M3 and R64 (12), code for proteins distantly homologous to each other (Table 1) and that belong to the VirB2 family, which forms the major T-pilus subunit in the A. tumefaciens VirB/D4 system (11). The product of *traY* is a distant homologue of DotA of L. pneumophila (37) and, together with ExcA, builds the entry exclusion system of R64 (42) and of pCTX-M3 (17). The putative functions of the other proteins encoded in the *tra* and *trb* regions of pCTX-M3 remain unknown (Table 1).

### Putative regulators of the pCTX-M3 transfer genes.

**FIG 5:**
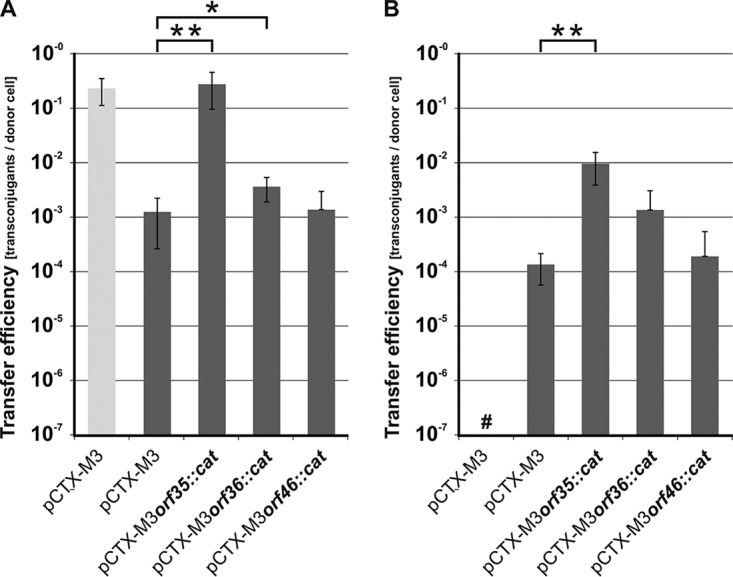
Mobilization efficiency of pToriT for different pCTX-M3 derivatives. Conjugative transfer efficiency of pCTX-M3 (light gray) and mobilization efficiencies of pToriT (dark gray) by pCTX-M3 or its deletion derivatives pCTX-M3*orf35*::*cat*, pCTX-M3*orf36*::*cat*, and pCTX-M3*orf46*::*cat* as helper plasmids into recipient E. coli (A) and A. tumefaciens (B) cells are shown. #, undetectable transfer (<10^−7^). Transconjugants with pCTX-M3 were selected on gentamicin; those with pToriT were selected on kanamycin. Each result is the mean from six experiments. Error bars indicate the SDs. *, *P* < 0.05; **, *P* < 0.01.

To identify the pCTX-M3 genes affecting the mobilization efficiency of plasmids bearing *oriT*_pCTX-M3_, we compared the ability of E. coli cells carrying pCTX-M3, pCTX-M3*orf35*::*cat*, pCTX-M3*orf36*::*cat*, or pCTX-M3*orf46*::*cat* plasmids to mobilize the pToriT plasmid into the recipient E. coli JE2571Rif^r^ cells (Fig. 5A). The helper plasmid pCTX-M3*orf35*::*cat* was >200-fold more effective in aiding pToriT mobilization than the other plasmids (2.76 × 10^−1^ versus 1.25 × 10^−3^, respectively; *P* = 0.0042). Surprisingly, pCTX-M3*orf36*::*cat* was approximately 5 times more effective than the WT plasmid (3.61 × 10^−3^ versus 1.25 × 10^−3^, respectively; *P* = 0.0124). The protein product of *orf35* of pCTX-M3 exhibits 44% amino acid sequence similarity with that of *yggA*, the first gene in the leading region of R64 (see Fig. S1), whose involvement in mobilization has not been studied.

With A. tumefaciens as the recipient, E. coli carrying pCTX-M3*orf35*::*cat* or pCTX-M3*orf36*::*cat* was >100- or 10-fold more efficient, respectively, as a pToriT donor than was E. coli bearing pCTX-M3 or pCTX-M3*orf46*::*cat* (Fig. 5B). However, only the increase in mobilization efficiency in the presence of pCTX-M3*orf35*::*cat* was statistically significant (*P* = 0.0024).

To further analyze the effects of *orf35* and *orf36* on plasmid mobilization, the deletions of these genes were complemented with appropriate plasmids, namely, pAL*orf35* and pAL*orf36*, respectively. The pABBoriT plasmid was mobilized to an E. coli recipient from E. coli DH5α donors. The helper plasmids pCS, pC35S, and pC36S, which were derived from pCTX-M3, pCTX-M3*orf35*::*cat*, and pCTX-M3*orf36*::*cat*, respectively, and lack kanamycin resistance, were generated for use with the Km^r^ pABBoriT plasmid (Table 2).

**TABLE 2:** Strains and plasmids used in the study

Strain or plasmid	Relevant feature or construction description^*a*^	Source or reference
Strains		
Escherichia coli		
DH5α	ϕ80 *lacZ*ΔM15 *deoR endA1 gyrA96 hsdR17 recA1 relA1 supE44 thi-1* Δ(*lacZYA argF*)*U169*	52
BW25113	Δ(*araD-araB*)*567* Δ(*rhaD-rhaB*)*568* Δ*lacZ4787*::*rrnB-3 hsdR514 rph-1*	27, 53
JE2571	*leu thr thi lacY thy pil fla*	25
JE2571Rif^r^	JE2571 selected on LB plus rifampin	This work
Agrobacterium tumefaciens LBA1010	Rif^r^	54
Pseudomonas putida KT2442	Rif^r^	55
Ralstonia eutropha JMP228	Rif^r^ *gfp* Km^r^	56
Plasmids		
pCTX-M3	IncM plasmid, 89,468 bp, Ap^r^ Pi^r^ Azt^r^ Caz^r^ Cft^r^ Km^r^ Gen^r^ To^r^	14, 15
pACYC184	Vector (*oriV*_P15A_ Tc^r^ Cm^r^)	57
pAL3	pUC18 BstUI fragment containing the *lacZ* gene and MCS, cloned into ScaI-PvuII pACYC184 (*oriV*_P15A_ Tc^r^)	This work
pBBR1 MCS-2	Vector (*oriV*_pBBR1_ *oriT*_RK2_ Km^r^)	58
pKD3	Template for generation of the PCR products used in gene disruption, *pir*-dependent replicon (*oriV*_R6Kγ_ Ap^r^ Cm^r^)	27
pKD46	Lambda Red recombinase expression plasmid, *repA101*(Ts) (*oriV*_R101_ Ap^r^)	27
pCP20	FLP recombinase expression plasmid, *repA101*(Ts) (*oriV*_R101_ Ap^r^ Cm^r^)	59
pMT5	pACYC184 SspI-MscI fragment containing a gene for Tc^r^ cloned into DraI-SspI pUC18 (*oriV*_pMB1_ Tc^r^)	This work
pUC18	Cloning vector (*oriV*_pMB1_ Ap^r^)	60
pABB20	Cloning vector (*oriV*_RA3_ Km^r^)	61
pOriT	*oriT*_pCTX-M3_ (nucleotides 31,616–31,721) cloned into the pUC18-derived pMI3 vector (*oriV*_pMB1_ Cm^r^)	14
pALoriT	pOriT EcoRI-PstI fragment containing *oriT*_pCTX-M3_ cloned into EcoRI-PstI pAL3 (*oriV*_p15A_ Tc^r^)	This work
pBBToriT	pALoriT XbaI-PvuI fragment containing the tetracycline resistance gene and *oriT*_pCTX-M3_ cloned into PvuI-XbaI pBBR1 MCS-2 (*oriV*_pBBR1_ Tc^r^)	This work
pToriT	pBBToriT derivative, fragment BsaI-Bst1107I with MOB_RK2_ removed (*oriV*_pBBR1_ Km^r^ Tc^r^)	This work
pABB20oriT	pOriT BamHI-PstI (blunted) fragment containing *oriT*_pCTX-M3_ cloned into BamHI-PstI pABB20 (*oriV*_RA3_ Km^r^)	This work
pHS11	pCTX-M3 derivative containing SexAI-SnaBI (nucleotides 36,645–40,568) and NruI-HindIII (nucleotides 51,663–58,653) fragments	This work
pCS	pCTX-M3 largest SalI fragment (nucleotides 1–595,520 and 79,940–89,468) self-ligated, Cft^r^	This work
pC35S	pCTX-M3*orf35*::*cat* largest SalI fragment self-ligated, Cft^r^ Cm^r^	This work
pC36S	pCTX-M3*orf36*::*ca*t largest SalI fragment self-ligated, Cft^r^ Cm^r^	This work

a Azt, aztreonam; Cft, cefotaxime; Caz, ceftazidime; Gen, gentamicin; Pi, piperacillin; To, tobramycin; (Ts), thermosensitive replication.

**FIG 6:**
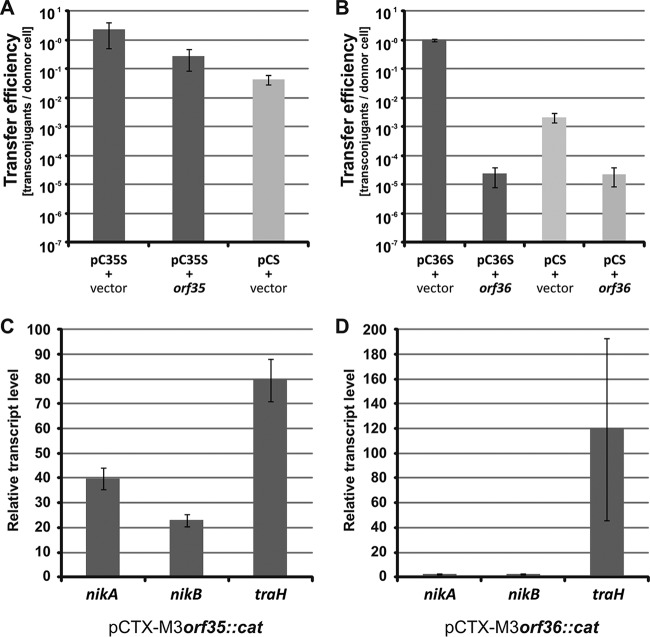
Effect of *orf35* and *orf36* on the efficiency of pABB20oriT mobilization. (A and B) Mobilization efficiency of pABB20oriT into E. coli recipient cells by respective helper plasmids: pCS (pCTX-M3 devoid of all antibiotic resistance genes except *bla*_TEM-1_ and *bla*_CTX-M-3_) and its derivatives pC35S (A) and pC36S (B) in the presence of pAL3 (vector), pAL*orf35* (*orf35*), or pAL*orf36* (*orf36*). (C and D) Relative transcript levels of *nikA*, *nikB*, and *traH* in E. coli strains bearing pCTX-M3 deletion derivatives pCTX-M3*orf35*::*cat* (C) or pCTX-M3*orf36*::*cat* (D). The transcript levels were normalized to those in cells bearing intact pCTX-M3. Each result is the mean from at least four experiments. Error bars indicate the SDs.

In the presence of pC35S and the complementing pAL*orf35* plasmid, the mobilization efficiency of pABB20oriT was slightly reduced relative to that observed in the presence of pC35S and the pAL3 vector (Fig. 6A), but this reduction occurred only when freshly obtained transformants were used as donors and the experiment was performed at 28°C. It is worth noting that the growth of E. coli bearing both pCTX-M3 and pAL*orf35* was disturbed, while the presence of pAL*orf35* alone did not affect cell growth. This effect can result from the possible deregulation of the *tra* genes controlled by the product of the *orf35* gene, especially when expressed from the two coresident plasmids.

The complementation of the *orf36* mutation in pC36S by pAL*orf36* decreased the pABB20oriT mobilization efficiency even below the level obtained with pCS as the helper plasmid (Fig. 6B). Interestingly, in the strain bearing both pCS and pAL*orf36*, the mobilization efficiency of pABB20oriT was reduced.

To address the question of the role of *orf35* and *orf36* in conjugative transfer, the transcript levels of the *nikA*, *nikB*, and *traH* genes, the first three genes of the *tra* operon, were determined in E. coli strains bearing either pCTX-M3*orf35*::*cat* or pCTX-*M3orf36*::*cat* and were compared with those in the control strain bearing intact pCTX-M3 (Fig. 6C and D). In the strain bearing pCTX-M3*orf35*::*cat*, the transcript levels of all three genes were elevated, approximately 40-, 23-, and 80-fold for *nikA*, *nikB*, and *traH*, respectively, relative to those in the control strain. In the strain bearing pCTX-M3*orf36*::*cat*, the levels of the *nikA* and *nikB* transcripts were unchanged, while the *traH* transcript was approximately 120-fold more abundant than in the control strain.

We propose that the pCTX-M3 *tra* operon, which encodes both the nickase complex and the T4SS, is subject to *orf35*-dependent repression. The effect of derepression of the *tra* operon in pCTX-M3*orf35*::*cat* cells would be visible for mobilizable multicopy plasmids, while the conjugation ability of the low-copy-number pCTX-M3*orf35*::*cat* would not benefit from the derepression of *tra* due to the limited number of accessible *oriT*-bearing plasmid molecules.

Similarly, the deletion of *orf36*, which is unique to IncL and IncM plasmids (14) and is dispensable for conjugation, increased the mobilization efficiency into E. coli and upregulated *traH* but not *nikA* or *nikB*. Moreover, the presence of additional copies of *orf36* significantly impaired mobilization even in donors bearing the native pCTX-M3 conjugative transfer region. We propose that the expression of *traH* and probably also that of the downstream genes encoding the T4SS are additionally regulated by the *orf36* product in a manner independent of *nikAB* transcription. The mechanism underlying the Orf35- and Orf36-dependent regulation is currently unknown and deserves further study, especially given that these predicted proteins do not contain known DNA-binding motifs (Table 1).

### Host ranges of the replicon and the conjugative transfer system of pCTX-M3.

Earlier studies (26) have demonstrated that pCTX-M3 can be transferred to A. tumefaciens via conjugation. Unexpectedly, despite a number of attempts, we were unable to transfer pCTX-M3 from E. coli to A. tumefaciens by mating (Fig. 5B). However, our mobilization experiments demonstrated that the conjugation system of pCTX-M3 is highly efficient in transferring the mobilizable broad-host-range (*oriV*_pBBR1_) plasmid pToriT, which contains *oriT*_pCTX-M3_, into A. tumefaciens (10^−4^ transconjugants per donor after 30 min of mating).

**FIG 7:**
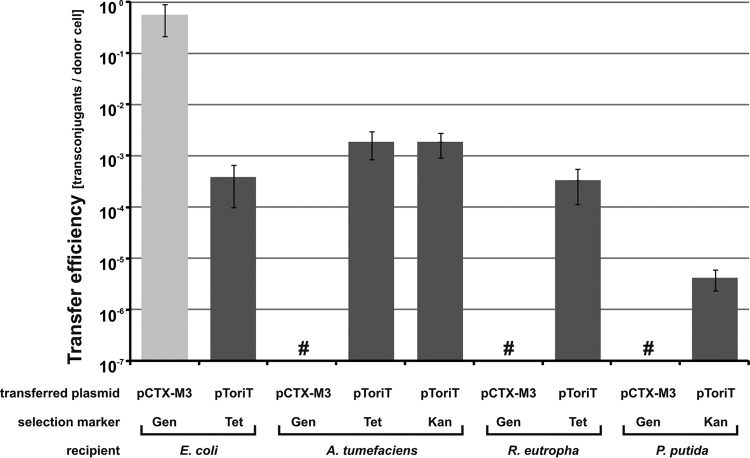
Host ranges of the pCTX-M3 replicon and conjugation system. Conjugative transfer efficiency of pCTX-M3 and mobilization efficiency of pToriT from E. coli DH5α(pCTX-M3, pToriT) into E. coli, A. tumefaciens, R. eutropha, and P. putida as recipients. Each result is the mean from at least three experiments. #, undetectable transfer (<10^−7^). Error bars indicate the SDs.

The finding that pCTX-M3 is not transferred into A. tumefaciens is inconsistent with previous results (26) showing that the conjugative transfer efficiency of the entire pCTX-M3 into *Alpha*-, *Beta*-, and Gammaproteobacteria was on the order of 10^−5^ transconjugants per donor cell after 24 h of mating. To investigate this discrepancy, we performed a 24-h mating experiment using E. coli DH5α(pCTX-M3, pToriT) as the donor and E. coli, A. tumefaciens, Ralstonia eutropha, and Pseudomonas putida as recipients. In such a system, the transfer of pCTX-M3 during mating reflects the host range of both its conjugation system and the IncM replicon, while the transfer of pToriT, the mobilizable broad-host-range plasmid containing *oriT*_pCTX-M3_, reflects the host range of the conjugation system of pCTX-M3 only. Transconjugants carrying pCTX-M3 were selected on plates containing gentamicin and rifampin, while those with pToriT were selected on plates with tetracycline and rifampin (A. tumefaciens, R. eutropha, and E. coli) or with kanamycin and rifampin (A. tumefaciens and P. putida). As shown in Fig. 7, the pCTX-M3 conjugation system is highly efficient in transferring pToriT into A. tumefaciens and R. eutropha (2 × 10^−3^ and 3 × 10^−4^ transconjugants per donor cell, respectively) and, with a lower efficiency, also into P. putida (3 × 10^−6^ per donor cell). In contrast, pCTX-M3 transconjugants were obtained only in the E. coli recipient. Thus, pCTX-M3 itself, when transferred, cannot be established in A. tumefaciens, R. eutropha, or P. putida. These results indicate that the host ranges of the pCTX-M3 conjugative transfer system and its replicon differ markedly; the former shows a broad range comprising *Alpha*-, *Beta*-, and Gammaproteobacteria, and the latter is restricted to Enterobacteriaceae. A similar observation concerning the differences between the host ranges of the conjugation system and the replicon has been reported previously for the narrow-host-range mobilizable Klebsiella pneumoniae plasmids pIGMS31 and pIGMS32 (43). These plasmids replicate only in Gammaproteobacteria, but their mobilization systems enable the conjugative transfer of a heterologous replicon into several Alphaproteobacteria hosts by the RK2 (IncP1α) conjugation system (43). In addition, recently, different host ranges of the conjugative and the replicative systems have been shown for the self-transferable P. putida plasmid NAH7 of the IncP-9 group (with the MPF_T_ system) (44).

### Concluding remarks.

Although the conjugation system of pCTX-M3 belongs to the MPF_I_ group, it differs from the one encoded by IncI1 plasmids. Therefore, it would be valuable to reevaluate the mobilization host range of MPF_I_ systems. It has been shown that ssDNA transiently generated during conjugative transfer triggers the SOS response in recipient cells unless the plasmid codes for an anti-SOS factor (45). As a consequence, homologous recombination and integron integrase genes are induced, leading to DNA rearrangements (45). Therefore, plasmids with an Mpf host range broader than their replicon host range, such as pCTX-M3, which does not code for an anti-SOS factor but does bear an integron, may have a greater impact on the adaptability of bacterial populations than previously appreciated.

## MATERIALS AND METHODS

### Bacterial strains, plasmids, and growth conditions.

The strains used in this work are listed in Table 2. E. coli DH5α was used as the host strain for DNA cloning. In the mating experiments, E. coli strain BW25113 or, where stated, strain DH5α bearing pCTX-M3 and its derivatives, was used as the donor, and E. coli strain JE2571Rif^r^ was used as the recipient. In transspecies matings, Pseudomonas putida, Ralstonia eutropha, or Agrobacterium tumefaciens was used as the recipient. The bacteria were cultured with agitation in LB medium (Biocorp, Warsaw, Poland) or on agar-solidified LB plates (46) at either 37°C (E. coli and P. putida) or 28°C (A. tumefaciens and R. eutropha). When required, antibiotics were added to the medium at the following final concentrations (μg/ml): ampicillin, 100; chloramphenicol, 20; gentamicin, 50; kanamycin, 50; rifampin, 100; tetracycline, 20 or 6 (for pToriT selection).

### Cloning and DNA manipulation.

Plasmid DNA was isolated by alkaline lysis using Plasmid Mini or Plasmid Midi kits (A&A Biotechnology, Gdańsk, Poland) according to the manufacturer's instructions. DNA cloning was performed according to standard protocols (46). All the enzymes used for cloning were from MBI Fermentas/Thermo Scientific (Vilnius, Lithuania).

### Plasmid constructions.

The plasmids that were constructed and used in this study are listed in Table 2 and Table S1 in the supplemental material. pCTX-M3 derivatives with deletions of genes from the *tra* and *trb* regions were constructed through lambda Red-mediated recombination (27). First, the BW25113(pKD46) strain was electrotransformed with pCTX-M3 and was selected on LB agar plates with gentamicin at 28°C. PCR products comprising the *cat* gene sequence with extensions homologous to the gene to be replaced on the pKD3 plasmid template were obtained using the primers listed in Table S2. Then, BW25113(pCTX-M3, pKD46) cells were electrotransformed with these DpnI-treated PCR products and were selected on LB agar plates with chloramphenicol at 37°C (to avoid the propagation of pKD46, which shows temperature-sensitive replication). Single colonies were isolated by the streak plate method at 37°C, and the correct integration of the *cat* gene into the target gene was verified by PCR (35 cycles) with the primer pairs listed in Table S3 and S4. The integration of *cat* in the four longest genes (*nikB*, *pri*, *traU*, and *traY*) was further analyzed by multiplex PCR with three primers (see Table S5): (i) catReVer, which anneals to the *cat* gene, (ii) a primer located upstream of the deleted gene (nikAF, priUVer, traUsU, or traYsU, respectively), and (iii) a primer that anneals to the gene to be deleted (nikBDVer, priDVer, traUDVer, or traYDVer, respectively). The primers were designed so that the expected products were smaller than 1 kb and enabled discrimination between the native pCTX-M3 and the appropriate mutant plasmid. All mutated plasmids were verified by sequencing with the catU142 primer. The loss of pKD46 was checked by multiplex PCR with the repKD46F and repKD46R primers, which were designed to amplify the *repA101* (thermosensitive replication) gene fragment, and with the TEMfor and TnTEMrev primers (for amplification of the *bla*_TEM-1_ gene, which is present in both pCTX-M3 and pKD46) as an internal PCR control (see Table S4). The constructed plasmid derivatives are listed in Table S1.

The *cat* gene was eliminated from six pCTX-M3 derivatives, namely, pCTX-M3*nikB*::*cat*, pCTX-M3*pri*::*cat*, pCTX-M3*traP*::*cat*, pCTX-M3*traU*::*cat*, pCTX-M3*traW*::*cat*, and pCTX-M3*traY*::*cat*, using pCP20, which encodes FLP recombinase, as a helper plasmid. In this process, the strain DH5α(pCP20) was electrotransformed with the appropriate pCTX-M3 derivative, and transformants were selected on LB agar with gentamicin at 28°C. Then, the transformants were streaked in parallel on LB with both chloramphenicol and gentamicin and were grown at 37°C. After colony purification, the clones that were chloramphenicol sensitive and gentamicin resistant were verified by PCR with the primers listed in Tables S3 and S5. The loss of pCP20 was verified by PCR with the repKD46F and repKD46R primers. The plasmids were then introduced into the BW25113 strain.

To construct the plasmids carrying individual genes from the *tra* and *trb* regions for use in the complementation experiments (Table S1), specific genes were amplified by PCR using *Pfu* DNA polymerase (Thermo Fisher Scientific, Waltham, MA) with the primers listed in Table S6 and were cloned into the pMT5 or pAL3 vectors, as described in Table S1. Genes which, probably due to the harmful effects of high-level expression, were not able to be cloned into the multicopy plasmid pMT5 (*oriV*_pMB1_), were cloned into the low-copy-number vector pAL3 (*oriV*_P15A_). Only the pAL*pri* plasmid was constructed without a PCR amplification step, as indicated in Table S1. The cloned genes were verified by sequencing (primers listed in Tables S3 and S4). The expression of the complementing genes cloned into the pAL3 and pMT5 vectors is driven by the lactose operon promoter (*P*_lac_).

Plasmids bearing *oriT*_pCTX-M3_ were obtained by cloning the *oriT* sequence into appropriate plasmids, as described in Table 2.

The plasmids pCS, pC35S, and pC36S (Table 2) were obtained by the digestion of pCTX-M3, pCTX-M3*orf35*::*cat*, and pCTX-M3*orf36*::*cat*, respectively, with SalI and the recircularization of the largest DNA fragment. Thus, these plasmids are devoid of all resistance genes except the *bla*_TEM-1_ and *bla*_CTX-M-3_ genes present in pCTX-M3.

### PCR conditions.

PCR was performed in a Veriti thermal cycler (Applied Biosystems, Foster City, CA) using Dream*Taq* or *Pfu* DNA polymerase with the supplied buffers (Thermo Fisher Scientific), deoxynucleoside triphosphate (dNTP) mixture, template DNA (purified DNA or bacterial colonies), and the appropriate primer pairs listed in Tables S2 to S6, according to the manufacturer's recommendations.

### DNA sequencing.

The sequencing was performed in the DNA Sequencing and Oligonucleotide Synthesis Laboratory at the Institute of Biochemistry and Biophysics, Polish Academy of Sciences, using a dye terminator sequencing kit and an automated sequencer (ABI 377; PerkinElmer, Waltham, MA).

### Real-time quantitative PCR.

RNA was isolated from cells of BW25113 strains bearing a specific pCTX-M3 deletion derivative alone or in combination with a plasmid carrying an appropriate complementing gene in the late exponential phase of growth (optical density at 600 nm [OD_600_] of 0.8 to 1) using a GeneJET RNA purification kit (Thermo Fisher Scientific) according to the manufacturer's protocol. RNA quality and integrity were checked by agarose gel electrophoresis, and the concentration was estimated using a NanoDrop ND-1000 spectrophotometer (Thermo Fisher Scientific). Three biological replicates were analyzed.

Reverse transcription was performed with random hexamer primers using the Maxima First Strand cDNA synthesis kit for RT-qPCR with dsDNase (Thermo Fisher Scientific). The specific qPCR primers used to amplify the reference (the *repA* gene encoding the replication initiator protein of the pCTX-M3 plasmid) and target genes are listed in Table S7. Real-time PCR was carried out using Real-Time 2×HS-PCR master mix SYBR (A&A Biotechnology) in a final volume of 10 μl in the LightCycler 480 system (Roche Life Sciences, Penzberg, Germany) with an initial denaturation at 95°C for 5 min followed by 40 cycles of amplification (95°C for 10 s and 50°C for 10 s). The relative gene expression in the deletion strains was calculated and normalized to the value obtained for a strain carrying the native pCTX-M3 plasmid (47).

### Plasmid conjugative transfer.

Cultures of the donor and recipient strains (approximately 10^8^ CFU ml^−1^) were grown in LB to stationary phase; the cultures were then washed twice with LB medium, and the donor strain was resuspended in a volume of LB equal to the initial volume of the culture, while the recipient strain was resuspended in one-fourth of the initial culture volume. Then, 0.5 ml of the donor and recipient suspensions was mixed and filtered through a sterile Millipore HA 0.45-μm filter (Millipore, Billerica, MA). The filter was incubated on an LB plate for 30 min at 37°C (E. coli) or 28°C (A. tumefaciens and E. coli). For the pCTX-M3 host range tests, the filter was incubated on an LB plate for 24 h at 37°C (P. putida and E. coli) or 28°C (A. tumefaciens and R. eutropha). Bacteria were washed from the filter with 1 ml of a sterile 0.85% NaCl solution. Conjugation was stopped by vigorously vortexing the mating mixture for 30 s and then placing it on ice. Serial dilutions of the mixture of donor, recipient, and transconjugant cells were plated on LB agar supplemented with the appropriate selection antibiotics. As a control, the donor and recipient cells were plated on LB supplemented with antibiotics for transconjugant selection. Mating in liquid was performed as described above but without the use of the filter: the mating mix was incubated for 30 min at 37°C, and conjugation was stopped by vortexing for 30 s. The conjugative transfer efficiency is equivalent to the number of transconjugants per donor cell.

### Bioinformatics analysis.

Nucleotide sequences were analyzed using Clone Manager 9 professional edition. Sequence similarity searches were performed using the BLAST programs (48) provided by the National Center for Biotechnology Information (NCBI) (http://blast.ncbi.nlm.nih.gov/Blast.cgi) to search against the NCBI “nr” (nonredundant) DNA or protein database with standard parameters; no filters or masks were applied. The molecular weight and theoretical pI were calculated with the use of the ProtParam tool of Expasy (http://web.expasy.org/protparam/) (48). Motifs in protein sequences were searched with the use of MotifScan (http://myhits.isb-sib.ch/cgi-bin/motif_scan). SignalP v.2.0 (http://www.cbs.dtu.dk/services/SignalP-2.0/) was used to search for signal peptides, TMPred (http://www.ch.embnet.org/software/TMPRED_form.html) was used to determine the presence of transmembrane helices (TMH) (49), and LipoP (http://www.cbs.dtu.dk/services/LipoP/) was used to predict lipid attachment motifs (50).

### Statistical analysis.

Data concerning the plasmid conjugative transfer frequencies are presented as the means ± standard deviations (SDs). The differences between the mobilization efficiencies of pToriT by the different plasmids (Fig. 5) were tested for statistical significance using the *t* test (Prism 6; GraphPad Software, Inc., La Jolla, CA). *P* values of <0.05 were considered statistically significant.

## Supplementary Material

Supplemental file 1

## References

[1] Lawley T, Klimke W, Gubbins M, Frost L 2003 F factor conjugation is a true type IV secretion system. FEMS Microbiol Lett 224:1–15. doi:10.1016/S0378-1097(03)00430-0. 12855161

[2] Christie PJ, Atmakuri K, Krishnamoorthy V, Jakubowski S, Cascales E 2005 Biogenesis, architecture, and function of bacterial type IV secretion systems. Annu Rev Microbiol 59:451–485. doi:10.1146/annurev.micro.58.030603.123630. 16153176 PMC3872966

[3] Christie PJ 2016 The mosaic type IV secretion systems. EcoSal Plus 2016. doi:10.1128/ecosalplus.ESP-0020-2015. PMC5119655 27735785

[4] Nagai H, Kubori T 2011 Type IVB secretion systems of *Legionella* and other Gram-negative bacteria. Front Microbiol 2:136. doi:10.3389/fmicb.2011.00136. 21743810 PMC3127085

[5] Grohmann E, Christie PJ, Waksman G, Backert S 2018 Type IV secretion in Gram-negative and Gram-positive bacteria. Mol Microbiol 107:455–471. doi:10.1111/mmi.13896. 29235173 PMC5796862

[6] Fronzes R, Christie PJ, Waksman G 2009 The structural biology of type IV secretion systems. Nat Rev Microbiol 7:703–714. doi:10.1038/nrmicro2218. 19756009 PMC3869563

[7] Cabezón E, Ripoll-Rozada J, Pena A, de la Cruz F, Arechaga I 2015 Towards an integrated model of bacterial conjugation. FEMS Microbiol Rev 39:81–95. 25154632 10.1111/1574-6976.12085

[8] Fronzes R, Schäfer E, Wang L, Saibil HR, Orlova EV, Waksman G 2009 Structure of a type IV secretion system core complex Science 323:266–268. doi:10.1126/science.1166101. 19131631 PMC6710095

[9] Bhatty M, Laverde Gomez JA, Christie PJ 2013 The expanding bacterial type IV secretion lexicon. Res Microbiol 164:620–639. doi:10.1016/j.resmic.2013.03.012. 23542405 PMC3816095

[10] Alvarez-Martinez CE, Christie PJ 2009 Biological diversity of prokaryotic type IV secretion systems. Microbiol Mol Biol Rev 73:775–808. doi:10.1128/MMBR.00023-09. 19946141 PMC2786583

[11] Guglielmini J, Bertrand N, Abby SS, Garcillan-Barcia MP, de la Cruz F, Rocha EP 2014 Key components of the eight classes of type IV secretion systems involved in bacterial conjugation or protein secretion. Nucleic Acids Res 42:5715–5727. doi:10.1093/nar/gku194. 24623814 PMC4027160

[12] Komano T, Yoshida T, Narahara K, Furuya N 2000 The transfer region of Incl1 plasmid R64: similarities between R64 *tra* and *Legionella icm*/*dot* genes. Mol Microbiol 35:1348–1359. doi:10.1046/j.1365-2958.2000.01769.x. 10760136

[13] Vincent CD, Friedman JR, Jeong KC, Buford EC, Miller JL, Vogel JP 2006 Identification of the core transmembrane complex of the *Legionella* Dot/Icm type IV secretion system. Mol Microbiol 62:1278–1291. doi:10.1111/j.1365-2958.2006.05446.x. 17040490

[14] Gołębiewski M, Kern-Zdanowicz I, Zienkiewicz M, Adamczyk M, Żylinska J, Baraniak A, Gniadkowski M, Bardowski J, Cegłowski P 2007 Complete nucleotide sequence of the pCTX-M3 plasmid and its involvement in spread of the extended-spectrum β-lactamase gene *bla*_CTX-M-3_. Antimicrob Agents Chemother 51:3789–3795. doi:10.1128/AAC.00457-07. 17698626 PMC2151408

[15] Gniadkowski M, Schneider I, Jungwirth R, Hryniewicz W, Bauernfeind A 1998 Ceftazidime-resistant *Enterobacteriaceae* isolates from three Polish hospitals: identification of three novel TEM- and SHV-5-type extended-spectrum β-lactamases. Antimicrob Agents Chemother 42:514–520. 9517925 10.1128/aac.42.3.514 PMC105491

[16] Bonnin RA, Nordmann P, Carattoli A, Poirel L 2013 Comparative genomics of IncL/M-type plasmids: evolution by acquisition of resistance genes and insertion sequences. Antimicrob Agents Chemother 57:674–676. doi:10.1128/AAC.01086-12. 23114767 PMC3535931

[17] Carattoli A, Seiffert SN, Schwendener S, Perreten V, Endimiani A 2015 Differentiation of IncL and IncM plasmids associated with the spread of clinically relevant antimicrobial resistance. PLoS One 10:e0123063. doi:10.1371/journal.pone.0123063. 25933288 PMC4416936

[18] Carattoli A 2013 Plasmids and the spread of resistance. Int J Med Microbiol 303:298–304. doi:10.1016/j.ijmm.2013.02.001. 23499304

[19] Poirel L, Bonnin RA, Nordmann P 2012 Genetic features of the widespread plasmid coding for the carbapenemase OXA-48. Antimicrob Agents Chemother 56:559–562. doi:10.1128/AAC.05289-11. 22083465 PMC3256075

[20] Espedido BA, Steen JA, Ziochos H, Grimmond SM, Cooper MA, Gosbell IB, van Hal SJ, Jensen SO 2013 Whole genome sequence analysis of the first Australian OXA-48-producing outbreak-associated *Klebsiella pneumoniae* isolates: the resistome and *in vivo* evolution. PLoS One 8:e59920. doi:10.1371/journal.pone.0059920. 23555831 PMC3612081

[21] Bryant KA, Van Schooneveld TC, Thapa I, Bastola D, Williams LO, Safranek TJ, Hinrichs SH, Rupp ME, Fey PD 2013 KPC-4 is encoded within a truncated Tn*4401* in an IncL/M plasmid, pNE1280, isolated from *Enterobacter cloacae* and *Serratia marcescens*. Antimicrob Agents Chemother 57:37–41. doi:10.1128/AAC.01062-12. 23070154 PMC3535906

[22] Dolejska M, Papagiannitsis CC, Medvecky M, Davidova-Gerzova L, Valcek A 2018 Characterization of the complete nucleotide sequences of IMP-4-encoding plasmids, belonging to diverse Inc families, recovered from *Enterobacteriaceae* of wildlife origin. Antimicrob Agents Chemother 62:e02434–17. doi:10.1128/AAC.02434-17. 29483121 PMC5923110

[23] González-Zorn B, Catalan A, Escudero JA, Domínguez L, Teshager T, Porrero C, Moreno MA 2005 Genetic basis for dissemination of *armA*. J Antimicrob Chemother 56:583–585. doi:10.1093/jac/dki246. 16027145

[24] Yoshida T, Furuya N, Ishikura M, Isobe T, Haino-Fukushima K, Ogawa T, Komano T 1998 Purification and characterization of thin pili of IncI1 plasmids ColIb-P9 and R64: formation of PilV-specific cell aggregates by type IV pili. J Bacteriol 180:2842–2848. 9603870 10.1128/jb.180.11.2842-2848.1998 PMC107247

[25] Bradley DE 1980 Determination of pili by conjugative bacterial drug resistance plasmids of incompatibility groups B, C, H, J, K, M, V, and X. J Bacteriol 141:828–837. 6102552 10.1128/jb.141.2.828-837.1980 PMC293694

[26] Mierzejewska J, Kulińska A, Jagura-Burdzy G 2007 Functional analysis of replication and stability regions of broad-host-range conjugative plasmid CTX-M3 from the IncL/M incompatibility group. Plasmid 57:95–107. doi:10.1016/j.plasmid.2006.09.001. 17087993

[27] Datsenko KA, Wanner BL 2000 One-step inactivation of chromosomal genes in *Escherichia coli* K-12 using PCR products. Proc Natl Acad Sci U S A 97:6640–6645. doi:10.1073/pnas.120163297. 10829079 PMC18686

[28] Furuya N, Komano T 1997 Mutational analysis of the R64 *oriT* region: requirement for precise location of the NikA-binding sequence. J Bacteriol 179:7291–7297. doi:10.1128/jb.179.23.7291-7297.1997. 9393692 PMC179678

[29] Guglielmini J, de la Cruz F, Rocha EP 2013 Evolution of conjugation and type IV secretion systems. Mol Biol Evol 30:315–331. doi:10.1093/molbev/mss221. 22977114 PMC3548315

[30] Segal G, Feldman M, Zusman T 2005 The Icm/Dot type-IV secretion systems of *Legionella pneumophila* and *Coxiella burnetii*. FEMS Microbiol Rev 29:65–81. doi:10.1016/j.femsre.2004.07.001. 15652976

[31] Kerr JE, Christie PJ 2010 Evidence for VirB4-mediated dislocation of membrane-integrated VirB2 pilin during biogenesis of the *Agrobacterium* VirB/VirD4 type IV secretion system. J Bacteriol 192:4923–4934. doi:10.1128/JB.00557-10. 20656905 PMC2944537

[32] Berger BR, Christie PJ 1993 The *Agrobacterium tumefaciens virB4* gene product is an essential virulence protein requiring an intact nucleoside triphosphate-binding domain. J Bacteriol 175:1723–1734. doi:10.1128/jb.175.6.1723-1734.1993. 8449880 PMC203967

[33] Narahara K, Rahman E, Furuya N, Komano T 1997 Requirement of a limited segment of the *sog* gene for plasmid R64 conjugation. Plasmid 38:1–11. doi:10.1006/plas.1997.1297. 9281491

[34] Chatfield LK, Wilkins BM 1984 Conjugative transfer of IncI 1 plasmid DNA primase. Mol Gen Genet 197:461–466. doi:10.1007/BF00329943. 6396492

[35] Rees CE, Wilkins BM 1989 Transfer of *tra* proteins into the recipient cell during bacterial conjugation mediated by plasmid ColIb-P9. J Bacteriol 171:3152–3157. doi:10.1128/jb.171.6.3152-3157.1989. 2656642 PMC210029

[36] Wilkins BM, Thomas AT 2000 DNA-independent transport of plasmid primase protein between bacteria by the I1 conjugation system. Mol Microbiol 38:650–657. doi:10.1046/j.1365-2958.2000.02164.x. 11069687

[37] Furuya N, Komano T 1996 Nucleotide sequence and characterization of the *trbABC* region of the IncI1 plasmid R64: existence of the *pnd* gene for plasmid maintenance within the transfer region. J Bacteriol 178:1491–1497. doi:10.1128/jb.178.6.1491-1497.1996. 8626273 PMC177830

[38] Vincent CD, Friedman JR, Jeong KC, Sutherland MC, Vogel JP 2012 Identification of the DotL coupling protein subcomplex of the *Legionella* Dot/Icm type IV secretion system. Mol Microbiol 85:378–391. doi:10.1111/j.1365-2958.2012.08118.x. 22694730 PMC3391322

[39] Sutherland MC, Binder KA, Cualing PY, Vogel JP 2013 Reassessing the role of DotF in the *Legionella pneumophila* type IV secretion system. PLoS One 8:e65529. doi:10.1371/journal.pone.0065529. 23762385 PMC3676331

[40] Furuya N, Nisioka T, Komano T 1991 Nucleotide sequence and functions of the *oriT* operon in Incll plasmid R64. J Bacteriol 173:2231–2237. doi:10.1128/jb.173.7.2231-2237.1991. 1848841 PMC207772

[41] Kuroda T, Kubori T, Thanh Bui X, Hyakutake A, Uchida Y, Imada K, Nagai H 2015 Molecular and structural analysis of *Legionella* DotI gives insights into an inner membrane complex essential for type IV secretion. Sci Rep 5:10912. doi:10.1038/srep10912. 26039110 PMC4454188

[42] Sakuma T, Tazumi S, Furuya N, Komano T 2013 ExcA proteins of IncI1 plasmid R64 and IncIγ plasmid R621a recognize different segments of their cognate TraY proteins in entry exclusion. Plasmid 69:138–145. doi:10.1016/j.plasmid.2012.11.004. 23201046

[43] Smorawinska M, Szuplewska M, Zaleski P, Wawrzyniak P, Maj A, Plucienniczak A, Bartosik D 2012 Mobilizable narrow host range plasmids as natural suicide vectors enabling horizontal gene transfer among distantly related bacterial species. FEMS Microbiol Lett 326:76–82. doi:10.1111/j.1574-6968.2011.02432.x. 22092700

[44] Kishida K, Inoue K, Ohtsubo Y, Nagata Y, Tsuda M 2017 Host range of the conjugative transfer system of IncP-9 naphthalene-catabolic plasmid NAH7 and characterization of its *oriT* region and relaxase. Appl Environ Microbiol 83:e02359–16. doi:10.1128/AEM.02359-16. 27742684 PMC5165122

[45] Baharoglu Z, Bikard D, Mazel D 2010 Conjugative DNA transfer induces the bacterial SOS response and promotes antibiotic resistance development through integron activation. PLoS Genet 6:e1001165. doi:10.1371/journal.pgen.1001165. 20975940 PMC2958807

[46] Sambrook J, Fritsch EF, Maniatis T 1989 Molecular cloning: a laboratory manual. Cold Spring Harbor Laboratory, Cold Spring Harbor, NY.

[47] Livak KJ, Schmittgen TD 2001 Analysis of relative gene expression data using real-time quantitative PCR and the 2-ΔΔ*C_T_* method. Methods 25:402–408. doi:10.1006/meth.2001.1262. 11846609

[48] Altschul S 1997 Gapped BLAST and PSI-BLAST: a new generation of protein database search programs. Nucleic Acids Res 25:3389–3402. doi:10.1093/nar/25.17.3389. 9254694 PMC146917

[49] Nielsen H, Engelbrecht J, Brunak S, von Heijne G 1997 Identification of prokaryotic and eukaryotic signal peptides and prediction of their cleavage sites. Protein Eng Des Sel 10:1–6. doi:10.1093/protein/10.1.1. 9051728

[50] Juncker AS, Willenbrock H, von Heijne G, Brunak S, Nielsen H, Krogh A 2003 Prediction of lipoprotein signal peptides in Gram-negative bacteria. Protein Sci 12:1652–1662. doi:10.1110/ps.0303703. 12876315 PMC2323952

[51] Thomas CM, Thomson NR, Cerdeño-Tárraga AM, Brown CJ, Top EM, Frost LS 2017 Annotation of plasmid genes. Plasmid 91:61–67. doi:10.1016/j.plasmid.2017.03.006. 28365184

[52] Hanahan D 1983 Studies on transformation of *Escherichia coli* with plasmids. J Mol Biol 166:557–580. doi:10.1016/S0022-2836(83)80284-8. 6345791

[53] Grenier F, Matteau D, Baby V, Rodrigue S 2014 Complete genome sequence of *Escherichia coli* BW25113. Genome Announc 2:e01038–14. doi:10.1128/genomeA.01038-14. 25323716 PMC4200154

[54] Koekman BP, Hooykaas PJJ, Schilperoort RA 1982 A functional map of the replicator region of the octopine Ti plasmid. Plasmid 7:119–132. doi:10.1016/0147-619X(82)90072-5. 6281832

[55] Franklin FC, Bagdasarian M, Bagdasarian MM, Timmis KN 1981 Molecular and functional analysis of the TOL plasmid pWWO from *Pseudomonas putida* and cloning of genes for the entire regulated aromatic ring meta cleavage pathway. Proc Natl Acad Sci U S A 78:7458–7462. 6950388 10.1073/pnas.78.12.7458 PMC349287

[56] Top EM, Holben WE, Forney LJ 1995 Characterization of diverse plasmids isolated from soil by characterization of diverse 2,4-dichlorophenoxyacetic acid-degradative plasmids isolated from soil by complementation. Appl Environ Microbiol 61:1691–1698. 7646006 10.1128/aem.61.5.1691-1698.1995 PMC167431

[57] Chang AC, Cohen SN 1978 Construction and characterization of amplifiable multicopy DNA cloning vehicles derived from the P15A cryptic miniplasmid. J Bacteriol 134:1141–1156. 149110 10.1128/jb.134.3.1141-1156.1978 PMC222365

[58] Kovach ME, Elzer PH, Steven Hill D, Robertson GT, Farris MA, Roop RM, Peterson KM 1995 Four new derivatives of the broad-host-range cloning vector pBBR1MCS, carrying different antibiotic-resistance cassettes. Gene 166:175–176. doi:10.1016/0378-1119(95)00584-1. 8529885

[59] Cherepanov PP, Wackernagel W 1995 Gene disruption in *Escherichia coli*: Tc^r^ and Km^r^ cassettes with the option of Flp-catalyzed excision of the antibiotic-resistance determinant. Gene 158:9–14. doi:10.1016/0378-1119(95)00193-A. 7789817

[60] Vieira J, Messing J 1982 The pUC plasmids, an M13mp7-derived system for insertion mutagenesis and sequencing with synthetic universal primers. Gene 19:259–268. doi:10.1016/0378-1119(82)90015-4. 6295879

[61] Bartosik AA, Markowska A, Szarlak J, Kulińska A, Jagura-Burdzy G 2012 Novel broad-host-range vehicles for cloning and shuffling of gene cassettes. J Microbiol Methods 88:53–62. doi:10.1016/j.mimet.2011.10.011. 22056795

