# Supplementary material for: Characteristics of the Conjugative Transfer System of the IncM Plasmid pCTX-M3 and Identification of Its Putative Regulators
Source: J Bacteriol. 2018 Aug 24;200(18):e00234-18. doi: 10.1128/JB.00234-18 (PMC6112013; doi:10.1128/JB.00234-18)
Supplement: Supplemental file 1 [file zjb999094865s1.pdf]

Supplementary Information

Fig S1. Alignment of predicted amino acid sequences of the Orf35 protein of pCTX-M3 and YggA of the R64 plasmid

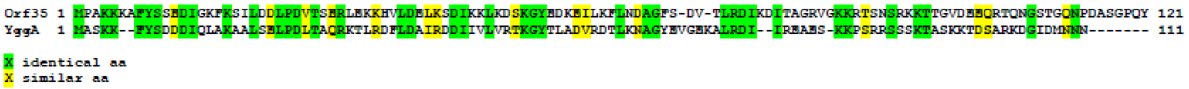

Table S1. pCTX-M3 plasmids with deletions of individual genes in the *tra* and *trb* regions and their respective complementing plasmids

Gene	pCTX-M3 derivative	Complementing plasmid	Construction details
excA	pCTX-M3*excA::cat*	pMT*excA*	pMT5 with PCR-amplified *excA* cloned into *Eco*RI-*Sma*I sites
nikA	pCTX-M3*nikA::cat*	pMT*nikA*	pMT5 with PCR amplified *nikA* cloned into *Eco*RI-*Sma*I sites
nikB	pCTX-M3*nikB::cat*<br>pCTX-M3Δ*nikB*	pMT*nikB*	pMT5 with PCR amplified *nikB* cloned into *Sac*I-*Sma*I sites
orf35	pCTX-M3*orf35::cat*	pAL*orf35*	pAL3 with PCR amplified *orf35* cloned into *Eco*RI-*Sma*I sites
orf36	pCTX-M3*orf36::cat*	pAL*orf36*	pAL3 with PCR amplified *orf36* cloned into *Eco*RI-*Sma*I sites
orf38	pCTX-M3*orf38::cat*	pMT*orf38*	pMT5 with PCR amplified *orf38* cloned into *Eco*RI-*Sma*I sites
orf46	pCTX-M3*orf46::cat*	none	
pri	pCTX-M3*pri::cat*<br>pCTX-M3Δ*pri*	pAL*pri*	pAL3 with *pri* from pHS11 (*Kpn*I-*Hin*cII) cloned into *Kpn*I-blunted *Pst*I sites
traH	pCTX-M3*traH::cat*	pMT*traH*	pMT5 with PCR amplified *traH* cloned into *Eco*RI-*Sma*I sites
traI	pCTX-M3*traI::cat*	pMT*traI*	pMT5 with PCR amplified *traI* cloned into *Eco*RI-*Sma*I sites
traJ	pCTX-M3*traJ::cat*	pMT*traJ*	pMT5 with PCR amplified *traJ* cloned into *Eco*RI-*Sma*I sites
traK	pCTX-M3*traK::cat*	pMT*traK*	pMT5 with PCR amplified *traK* cloned into *Eco*RI-*Sma*I sites
traL	pCTX-M3*traL::cat*	pMT*traL*	pMT5 with PCR amplified *traL* cloned into *Eco*RI-*Sma*I sites
traM	pCTX-M3*traM::cat*	pMT*traM*	pMT5 with PCR amplified *traM* cloned into *Eco*RI-*Sma*I sites
traN	pCTX-M3*traN::cat*	pMT*traN*	pMT5 with PCR amplified *traN* cloned into *Eco*RI-*Sma*I sites
traO	pCTX-M3*traO::cat*	pMT*traO*	pMT5 with PCR amplified *traO* cloned into *Sac*I-*Sma*I sites
traP	pCTX-M3*traP::cat*	pMT*traP*	pMT5 with PCR amplified *traP* cloned into *Eco*RI-*Sma*I sites
traQ	pCTX-M3*traQ::cat*	pMT*traQ*	pMT5 with PCR amplified *traQ* cloned into *Sac*I-*Sma*I sites
traR	pCTX-M3*traR::cat*	pMT*traR*	pMT5 with PCR amplified *traR* cloned into *Eco*RI-*Sma*I sites
traU	pCTX-M3*traU::cat*<br>pCTX-M3Δ*traU*	pAL*traU*	pAL3 with PCR amplified *traU* cloned into *Sac*I-*Sma*I sites
traW	pCTX-M3*traW::cat*	pAL*traW*	pAL3 with PCR amplified *traW* cloned into *Sma*I site
traX	pCTX-M3*traX::cat*	pAL*traX*	pAL3 with PCR amplified *traX* cloned into *Eco*RI-*Pst*I sites
traY	pCTX-M3*traY::cat*<br>pCTX-M3Δ*traY*	pAL*traY*	pAL3 with PCR amplified *traY* cloned into *Eco*RI-*Sma*I sites
trbA	pCTX-M3*trbA::cat*	pAL*trbA*	pAL3 with PCR amplified *trbA* cloned into *Eco*RI-*Sma*I sites
trbB	pCTX-M3*trbB::cat*	pMT*trbB*	pMT5 with PCR amplified *trbB* cloned into *Eco*RI-*Sma*I sites
trbC	pCTX-M3*trbC::cat*	pAL*trbC*	pAL3 with PCR amplified *trbC* cloned into *Eco*RI-*Sma*I sites
trbN	pCTX-M3*trbN::cat*	pMT*trbN*	pMT5 with PCR amplified *trbN* cloned into *Eco*RI-*Sma*I sites

Table S2. Primers used for generation of *cat* integration cassettes into genes from *tra* and *trb* regions of pCTX-M3

Gene	Primer	Sequence 5'-3'
excA	excAupP1	AGATAAGAGCAAGGACGGCATCAAAGGAGCTTAAA**TGTGTAGGCTGGAGCTGCTTCG**
	excAdnP2	CATATTCGGCGGGTTAAACTCAATAATTGCTGTTTA**CATATGAATATCCTCCTTA**
nikA	nikAupP1	CAAGCCCCGACACCCCCTAACGAGGTTAGCTATCTG**GTGTAGGCTGGAGCTGCTTCG**
	nikAdnP2	CTTTTTGTCCCTTCGGCCCTCAATGATTTTAGGTAT**CATATGAATATCCTCCTTA**
nikB	nikBupP1	CAGAAACTGCAACAGGAGATGGATGGTGATACCTAA**GTGTAGGCTGGAGCTGCTTCG**
	nikBdnP2	AACGTTTGCATTTTGATTTTCATTTCTTACTCCATTT**ATATGAATATCCTCCTTA**
orf35	orf35uP1	ATGGCATGGTCGATTTTGACATTATTGGGAGGGCA**TGTGTAGGCTGGAGCTGCTTCG**
	orf35dP2	ATAATGTGGGATGCGATATGCGAAAGGTGAAGGTTAAT**ATATGAATATCCTCCTTA**
orf36	orf36uP1	ATGCAAACAGTGATGCATTCCCGTTCCATTTGTAAC**GTGTAGGCTGGAGCTGCTTCG**
	orf36dP2	GAACAATGAGGTATACATGAGCGAACATAATGATTAT**ATATGAATATCCTCCTTA**
orf38	orf37uP1	GGCCCCGCCTTGCGGGGCCAATATCGGAGTTCCCTC**GTGTAGGCTGGAGCTGCTTCG**
	orf37dP2	ATCACATCCTCAAAGAAAGAAATCATTTAAAACCTCT**ATATGAATATCCTCCTTA**
orf46	orf46uP1	GGCGTTTGCCGGCGTTGTTAATCAGGAGGCCAAACG**GTGTAGGCTGGAGCTGCTTCG**
	orf46dP2	GGGGGACCACTCCCCGCCTTTTAACACAAACCTTCAT**ATATGAATATCCTCCTTA**
pri	priUpP1	TCTCAGCGATAACACATAACTACAGGGGTAGACAG**TGTGTAGGCTGGAGCTGCTTCG**
	priDnP2	AGTCTTTGCGACTGGTGCATGAGTCTGTTCTCTCCTT**ATATGAATATCCTCCTTA**
traH	traHupP1	ACGACAAATTCAATCCGAAGTAAAATGGAGTAAGAA**GTGTAGGCTGGAGCTGCTTCG**
	traHdnP2	CGGTAACGTCAGAAGCACGATCAGTTTAAGTTTCAT**CATATGAATATCCTCCTTA**
traI	traIupP1	AAGAAAGGTTCATTCCTCAGAGGGACCCGCCCATGA**GTGTAGGCTGGAGCTGCTTCG**
	traIdnP2	TTGGACCAAAATTGAATGCTTCCATCTACTGCCCCTT**ATATGAATATCCTCCTTA**
traJ	traJupP1	GCAGCTGGAAACCGACCATACGTTAAGGGGCAGTAG**GTGTAGGCTGGAGCTGCTTCG**
	traJdnP2	GCATAACGCCATTTGTTAATGTCCATACTTGCCCTCT**ATATGAATATCCTCCTTA**
traK	traKupP1	TATCTGGCGTTAAAAGAGGGCAAGTATGGACATTAA**GTGTAGGCTGGAGCTGCTTCG**
	traKdnP2	AGGTTTCGCCATACTGTCTACCCCTGTAGTTATGTGT**ATATGAATATCCTCCTTA**
traL	traLupP1	ATGCACCAGTCGCAAAGTCAGCGCCTCGGCCATAAA**GTGTAGGCTGGAGCTGCTTCG**
	traLdnP2	CTGTTTGCATCCTCACCACTTCCACTCGCAGCTGCCT**ATATGAATATCCTCCTTA**
traM	traMupP1	TTATGTTCGCTCATGTATACCTCATTGTTCATTAAC**GTGTAGGCTGGAGCTGCTTCG**
	traMdnP2	AGTACAGTTAACAGTTTAATCTTCATGATTGACCTCT**ATATGAATATCCTCCTTA**
traN	traNupP1	GGCCATTCTCAAACCCCGGAACTACTGAGGTCAATC**GTGTAGGCTGGAGCTGCTTCG**
	traNdnP2	TTCTTTACCTTTATCTGTAGCCAGCGTCGTTCCCCTT**ATATGAATATCCTCCTTA**
traO	traOupP1	TGACGTTGAAGTTGAGCTGAATTAAGGGGAACGACG**GTGTAGGCTGGAGCTGCTTCG**
	traOdnP2	ATTGTCATCTTCAAACAGATTCATTATCAATCTCCTT**ATATGAATATCCTCCTTA**
traP	traPupP1	CACCAATAACCCGTATTACCGTTAAGGAGATTGATA**GTGTAGGCTGGAGCTGCTTCG**
	traPdnP2	ATTCTAAATCCATAGTTTCGCCTTTTTAGGTCCTGTT**ATATGAATATCCTCCTTA**
traQ	traQupP1	CCTTATCCGCTAAACAGGACCTAAAAAGGCGAAACT**GTGTAGGCTGGAGCTGCTTCG**
	traQdnP2	ATAAGCTTAACAATTTTCATAAAGTATCCTTTTTGAT**ATATGAATATCCTCCTTA**
traR	traRupP1	CATTAAACATTCACTGGTAATCAAAAAGGATACTTT**GTGTAGGCTGGAGCTGCTTCG**
	traRdnP2	ATACCACTAACGATTTTGCCACCGTTCCGCGCAGCTC**ATATGAATATCCTCCTTA**
traU	traUupP1	TGAAATTTACAAGGGTAAGAGAGGTTTTAAATGATT**GTGTAGGCTGGAGCTGCTTCG**
	traUdnP2	TTTTGCTTTTCCGATTTTTATCATCTGAATCCCCTTT**ATATGAATATCCTCCTTA**
traW	traWupP1	AATCATTTCTGATGTGAGAAGGGGATTCAGATGATA**GTGTAGGCTGGAGCTGCTTCG**
	traWdnP2	CTGTTTTATTTTCGTCGGTCATTTTCTGGATACTCCT**ATATGAATATCCTCCTTA**
traX	traXupP1	GGAGAGTGTGAACGCGGGAGTATCCAGAAAATGACC**GTGTAGGCTGGAGCTGCTTCG**
	traXdnP2	AAGCAACCTTTTTAATTTACTCATGTGGCCCCCGCTT**ATATGAATATCCTCCTCA**
traY	traYupP1	CGCAGGCTGGTCAAGAGCGGGGGCCACATGAGTAAA**GTGTAGGCTGGAGCTGCTTCG**
	traYdnP2	GTCAGTGTTTGATAACGCATTCATATTTAAGCTCCTT**ATATGAATATCCTCCTTA**
trbA	trbAupP1	ATCGGGAAGGTTCACCAAAATTCTCTGAGGTTCTGA**GTGTAGGCTGGAGCTGCTTCG**
	trbAdnP2	CGATCGGTTTTTACGGTGAGTTTCATACTTCCTCGCT**ATATGAATATCCTCCTTA**
trbB	trbBupP1	CCCGTCCCGATCACCTGGAACTCTAAGCGAGGAAG**TGTGTAGGCTGGAGCTGCTTCG**
	trbBdnP2	GCAGGCGGGCATCGTCTTTGACGACGGAAACCGGGA**CATATGAATATCCTCCTTA**
trbC	trbCupP1	CTGCAGCAGGCAATAGGAGAATAACCCGGAGCCGC**TGTGTAGGCTGGAGCTGCTTCG**
	trbCdnP2	CTGATTAACAACGCCGGCAAACGCCGGCGTTCAGGGT**ATATGAATATCCTCCTTA**
trbN	trbNupP1	TTTTTTACTCGAAATAGAAATCATTAGGAGATTGT**TGTGTAGGCTGGAGCTGCTTCG**
	trbNdnP2	CCCTTTCCGTTGTGGCTGGTTTTGCATCAGAACCTCT**ATATGAATATCCTCCTTA**

Sequences homologous to the *cat* gene are shown in bold

Table S3. Primers used for verification of *cat* integration and *cat* elimination

Gene	Primer	Sequence 5'-3'	Expected product size (bp)		
			WT plasmid template	mutated plasmid template	
				with *cat* insertion	with *cat* eliminated
excA	excAsU<br>excAsD	CAGTGTTGGGTGATATGCAG<br>CCGTAAGGTTATGTAAACGC	1191	1567	
nikA	nikAsD<br>oriTminU	TCAGGAGTAGGCTGAACTGT<br>ATAGGATCCAGTACGGGACAAGATGTGTT	542	1246	
nikB	nikBsU<br>nikBsD	CGGAGTTCATAGCAAGGAGT<br>CCTTTTCGCTTTGAGAGGCA	2149	1197	267
orf35	orf35sU<br>oriTminD	GCTATATGCGAAAGGTGAAGG<br>TAACTGCAGAGATAGCTAACCTCGTTAGG	670	1320	
orf36	orf36sU<br>pCTX96	GGATGAGGTATGCAATACGG<br>CCGAGTCAGTTTGATCCATA	928	1368	
orf38	orf38sU<br>orf38sD	CTGACCGCATGGATCTCTTC<br>CCTGCGACGTAAGCCCTACA	702	1235	
orf46	orf46sU<br>pCTX101	ACGACGTGCTTTACCACCAG<br>ACCCGGAATAGGGTATACTG	980	1487	
pri	priSu<br>CTX13200	GCCATGATGGGCTACACGTT<br>TATAGGCAGCATCAGCACCAGC	3461	1313	389
traH	traHsU<br>traHsD	GAAACATCGTTTGATTCGTG<br>TGGCGGGTCCATGTAGTAAT	706	1223	
traI	traIsU<br>traIsD	CAACTGGCTAAACAGCGTGG<br>CGCCCTGCAACAGAATATCG	1117	1359	
traJ	traJsU<br>traJsD	TTAAGGCTCCAGTCATCACA<br>GGTAACGTGTAGCCAATCAT	1482	1353	
traK	traKsU<br>traKsD	TCGACGACCTGTTGATCGCT<br>TCTTTCAGCCGGCTCAGGTT	599	1363	
traL	traLsU<br>traLsD	CACGGATACGGCCATGTTGA<br>ACCGCCGCGTTGAAATACGA	765	1363	
traM	traMsU<br>traMsD	TGGCTTTTCGTCGTTCGCAC<br>GTGACTGCTGATTTACGGGC	1028	1285	
traN	traNsU<br>traNsD	ACTTTGCCAACCGTACAATG<br>TGAAGCTATCCCCTGTGAGA	1828	1691	
traO	traOsU<br>traOsD	GCCAGGGAGTACCAAAGTCT<br>CCACCAATCCATGCATAGTC	1717	1384	
traP	CTX17550<br>traPsD	TGCAACCTCAGCAGATGCCTCAGC<br>GCAGGCTCATCAAGTATCCA	970	1281	301
traQ	traQsU<br>traQsD	AGTCAACTTGGCGGAGTCAC<br>CAGCGGCAATACCACTAACG	753	1238	
traR	CTX18700<br>CTX19450	AAAGACTCTTACCTTGAGGGCC<br>ATTTTCTCCGGATGTTCGAGCG	750	1706	
traU	traUsU<br>traUsD	TGTCGCTTCTGTTGCAGAGT<br>GCGTTGAGATGGGAATGCTG	3246	1233	
traW	traWsU<br>traWsD	CCCTATGGAAGTGCATCAA<br>AAGAGCAGGCTGAATGTTTG	1534	1365	
traX	traXsU<br>traXsD	CAAACAGCAGCTGGAGAAAC<br>CATTTGACGCGATAGGTCAC	892	1281	
traY	traYsU<br>traYsD	CGCCGAAGTTATGAAACGGA<br>GCACTGCATCGATCACAAAG	2777	1629	699
trbA	5c25<br>3c56n	TTCAAAGCGGAACAGCTGCACCGC<br>ACTTTTGCGCCTCTGCAATGGC	1667	1455	
trbB	trbBsU<br>pCTX103	GACACCCCGAACAACAGTCT<br>AGGAACTGAGCAAACGCTAT	1829	1948	
trbC	trbCsU<br>trbCsD	GTTGCCGATTCGGGTGATGT<br>GGAGCCAGGAACGAGAAGTA	2299	1225	
trbN	trbNsU<br>trbNsD	CATTGTCTCTGTCGAAGGCA<br>GACAGGCTCCGTAAATCACA	648	1274	

Table S4. Primers used for verification of the presence of helper plasmids (pKD46 and pCP20) and for sequencing

Primer	Sequence 5'-3'	Application
**repKD46F**	TTTGCGTGAGCCATGAGAAC	pKD46 and pCP20 *repA101* detection - 774 bp
**repKD46R**	GGAAAGAACGGACGGTATCG	
**TEMfor**	CTGGATCTCAACAGCGGT	detection of $bla_{\mathrm{TEM-1}}$ in pKD46 and pCTX-M3 - 730 bp (1)
**catU142**	GTCGGCAGATGCTTAATG	sequencing primer
**traKsta**	ATGGACATTAACAAATGGCG	sequencing primer
**TnTEMrev**	CTGACAGTTACCAATGCT	(1)
**pri1F**	TGAATTTACGGCGTGGCTTG	pAL*pri* sequencing primer
**pri2F**	AGCAGCCAGTATCAGCGAAG	
**pri3F**	AGCTGCCGAACCGGAATACG	
**pri4F**	GAATGGAATGCGCTGGGAA	
**pri5F**	ACAGCCAACAGCAGGGAATG	

Table S5. Primers used for additional multiplex PCR verification of *cat* elimination

Gene	Primer	Sequence 5'-3'	Expected product size (bp): with *cat* insertion	Expected product size (bp): with *cat* eliminated
nikB	nikBDVer	GCTTACGGGTCAGGTAATTG	402	732
	nikAF	CCATGAAGCAGGGCTTAGTC		
	catReVer	GCTGGTGATATGGGATAGTG		
pri	priDVer	GCTTTACCGTCCACTGTATC	476	638
	priUVer	CTGCTGGCCATGATGGGCTACAC		
	catReVer	GCTGGTGATATGGGATAGTG		
traU	traUsU	TGTCGCTTCTGTTGCAGAGT	286	589
	traUDVer	ATGATTTAGCGCCCTGAACCTC		
	catReVer	GCTGGTGATATGGGATAGTG		
traY	traYsU	CGCCGAAGTTATGAAACGGA	424	545
	traYDVer	GGCGACGCCACAACGAAAAGAGATAG		
	catReVer	GCTGGTGATATGGGATAGTG		

Table S6. Primers used for PCR-amplified gene cloning intopAL3 and pMT5 vectors

Gene	Primer	Sequence 5'-3'
excA	excAUEc	GATGAATTCAAGGACGGCATCAAAGGAGC
	excAsD	CCGTAAGGTTATGTAAACGC
nikA	nikAUEc	CCCCGAATTCCCCTAACGAGGTTAGC
	nikAsD	TCAGGAGTAGGCTGAACTGT
nikB	nikBUSa	AGGAGCTCCAACAGGAGATGGATGG
	nikBsD	CCTTTTCGCTTTGAGAGGCA
orf35	orf35UEc	TCGAATTCGACATTATTGGGAGGGC
	orf35sU	GCTATATGCGAAAGGTGAAGG
orf36	orf36UEc	TGGAATTCAACAATCCGGCAGCTGCGAGTGG
	pCTX96	CCGAGTCAGTTTGATCCATA
orf38	orf38UEc	CCGAATTCCGGGGCCAATATCGGAGTTC
	orf38sD	CCTGCGACGTAAGCCCTACA
traH	traHUEc	TTGAATTCGAAGTAAAATGGAGTAAG
	traHDKp	GCGGTACCGTCAGAAGCACGATCAG
traI	traIUEc	AAGAATTCATTCCTCAGAGGGACCC
	traIsD	CGCCCTGCAACAGAATATCG
traJ	traJUEc	GAGAATTCGTTAAGGGGCAGTAGATG
	traJDKp	ATGGTACCATTTGTTAATGTCCATACTTGC
traK	traKUEc	CTGGAATTCAAAGAGGGCAAGTATGGAC
	traKsD	TCTTTCAGCCGGCTCAGGTT
traL	traLUEc	AAGAATTCGAAAGGAGAGAACAGACTC
	traLsD	ACCGCCGCGTTGAAATACGA
traM	traMUEc	CAGAATTCCTGCACAGGATTCGTAATC
	traMDKp	GCGGTACCATAAGTACAGTTAACAG
traN	traNUEc	GGGAATTCTCAAACCCCGGAACTACTG
	traNDKp	TCGGTACCTTTATCTGTAGCCATCG
traO	traOUSa	TTGAGCTCAATTAAGGGGAACGAC
	traOsD	CCACCAATCCATGCATAGTC
traP	traPUEc	CCGAATTCCCGTTAAGGAGATTGATAATG
	traPDKp	CTGGTACCATAGTTTCGCCTTTTTAGG
traQ	traQUSa	TATGAGCTCAACAGGACCTAAAAAGGC
	traQsD	CAGCGGCAATACCACTAACG
traR	traRUEc	TAAGAATTCACTGGTAATCAAAAAGGATAC
	CTX19450	ATTTTCTCCGGATGTTCGAGCG
traU	traUUSa	GGGAGCTCATTTACAAGGGTAAGAGAG
	traUsD	GCGTTGAGATGGGAATGCTG
traW	traWUMu	ATCAATTGTGATGTGAGAAGGGGATTCAGATG
	traWDKp	TTGGTACCGGTCATTTTCTGGATACTCCC
traX	traXUEc	CTGAATTCTGTGAACGCGGGAGTATCCAG
	traXsD	CATTTGACGCGATAGGTCAC
traY	traYUEc	TTAAGAATTCGCAGGCTGGTCAAGAGCGG
	traYsD	GCACTGCATCGATCACAAAG
trbA	trbAUEc	CCAGAATTCTCTGAGGTTCTGATG
	5c25	TTCAAAGCGGAACAGCTGCACCGC
trbB	trbBuEc2	CCGAATTCCAGTCTGCGACTGACAGACGATG
	trbBDKp	GCGGTACCGGGTTATTCTCCTATTGC
trbC	trbCUEc	AGAATTCAATAGGAGAATAACCCG
	trbCsD	GGAGCCAGGAACGAGAAGTA
trbN	trbNUEc	TAGAATTCATTAGGAGATTGTTATGGTTG
	trbNDKp	CCGGGTACCGTTGTGGCTGGTTTTG

Relevant restriction sites are underlined

Table S7. Primers for RT-qPCR

Gene	Primer	Sequence 5'-3'
repA	pRepAaF<br>pRepAaR	TCACATGGGACCCGTTTAACC<br>GCGCGGCTTTAACAGAGATTTC
nikA	pCNikAaF<br>pCNikAaR	TCTTATCAAATCTGGGCTTGGAAAG<br>ACCATCCATCTCCTGTTGCAG
nikB	pCNikBaF<br>pCNikBaR	GAAAGAATGCTGCTCAAGGGG<br>CGTTTGAACTCAGCCAGGTTG
traH	pCTraHaF<br>pCTraHaR	CCCGTTACAAATTCCCTACC<br>GCAACTGTTCAAACGTAACC
orf35	pCOrf5aF<br>pCOrf5aR	AAGCATTCTCGACGACTTG<br>CCGGCAGTAATGTCCTTG
orf36	pCOrf3aF<br>pCOrf3aR	TAGCTTGCCGGTTCGTATTTC<br>CCGAGATGAGCGACGATGAG
traL	pCtraLaF<br>pCtraLaR	GGGAGCCTGACTAATACAAC<br>ATGGAACGGGAATGCATCAC
traM	pCtraMaF<br>pCtraMaR	TTCAGGGTTACTGGCTTCAC<br>CCGCGGACGCTGGTTAATTG
traN	pCtraNaF<br>pCtaNaR	GCAGACACTGGAAGAGTTAC<br>CCGTACCTGCGGAATGGATG
traO	pCtraOaF<br>pCTraOaR	AGATAAAGCGGCTCTCACAG<br>ACGTAGCCACCGCTTTAGCC
traP	pCtraPaF<br>pCtraPaR	GGCAGGATTCTGGTGACTTC<br>GCCCATAACCAGCGGCTTTC
traQ	pCtraQaF<br>pCtraQaR	GCAGTCGTTGGGATCATTGG<br>CTGACGGGTCCGAAGCTAAC
traU	pCtraUaF<br>pCtraUaF	GCCGTGAACGCGCTTACATC<br>CAATCAGGCGCCACAGCATC
traW	pCtraWaF<br>pCtraWaR	GCAGGCACAGGATAAGTACC<br>AACTTCGGCCGTTGAAGATG
traX	pCtraXaF<br>pCtraXaR	CCCGGAAGGGAAGGCTAAAC<br>TTCATAACTTCGGCGAACGG
traY	pCtraYaF<br>pCtraYaR	TGGTGCGGTTCAATCATTGG<br>TGGCCAGAAGCCTCCATTTG
trbN	pCTrbNaF<br>pCTrbNaR	GTGGATTCCTCGCCTTGTGG<br>TGTTGTATTTGAGGATGTAGGCAC
excA	pCexcAaF<br>pCexcAaR	TGGACGGTTAGAGACGGTTC<br>TTCTCCGGGCCAGGTCATAG

References

1. **Avison MB**, **von Heldreich CJ**, **Higgins CS**, **Bennett PM**, **Walsh TR**. 2000. A TEM-2 β-lactamase encoded on an active Tn*1*-like transposon in the genome of a clinical isolate of *Stenotrophomonas maltophilia*. J Antimicrob Chemother **46**: 879–884.
